# Thinking, Feeling, and Acting with Generative AI: Affective, Behavioral, and Cognitive Engagement and Perceived Human–AI Outcomes

**DOI:** 10.3390/jintelligence14100267

**Published:** 2026-10-16

**Authors:** Amir Mahmood, Chaocheng Zhou, Xiao Huang, Nadia Rehman, Misbah Shafait Abbasi, Akpaasou Daniel Terver

**Affiliations:** 1 College of Education, Zhejiang Normal University, Jinhua 321004, China; amirmahmood@zjnu.edu.cn (A.M.); misbah@zjnu.edu.cn (M.S.A.); danielakpaasou@zjnu.edu.cn (A.D.T.); 2 School of Public Affairs, Zhejiang Shuren University, Hangzhou 310015, China; cczhou@126.com; 3 The Joint Education Institute of Zhejiang Normal University and the University of Kansas, Zhejiang Normal University, Jinhua 321004, China

**Keywords:** AI engagement, generative AI, affective–behavioral–cognitive model, SEM, trust calibration, creativity, integrity

## Abstract

Generative AI requires students not only to adopt technology but also to engage critically, behaviorally, cognitively, and emotionally with its outputs. This study develops and tests the AI Engagement ABC+ Model, a domain-specific adaptation of the affective–behavioral–cognitive engagement framework. The model differentiates positive and negative AI affect, three behavioral functions, creation, verification, and community participation, and two cognitive regulatory functions, strategic and critical cognition. A cross-sectional online survey collected data from 1250 students representing 15 nationalities at eight universities in China. Partial least squares structural equation modeling was used to examine 15 hypothesized associations between seven first-order engagement facets and four self-reported AI-supported outcomes: perceived Output Quality, Creativity, Integrity, and Trust Calibration. Fourteen hypotheses were supported. Verification Behavior had the largest estimated coefficient among the included predictors of Integrity (β = 0.303) and perceived Output Quality (β = 0.269) and was also associated with perceived Trust Calibration (β = 0.191). Critical Cognition had the largest estimated coefficient among the included predictors of perceived Trust Calibration (β = 0.270), and Community Behavior was positively associated with Integrity (β = 0.290) and Creativity (β = 0.240). Positive AI Affect was associated with perceived Output Quality (β = 0.214) and Creativity (β = 0.139), whereas Negative AI Affect was negatively associated with Trust Calibration (β = −0.137) but was unrelated to Integrity. The model explained 25.7% of the variance in Creativity, 25.2% in Integrity, 20.8% in perceived Output Quality, and 17.6% in Trust Calibration. Positive Q^2^_predict values provided additional evidence of out-of-sample predictive relevance. The findings support a differentiated view of GenAI engagement in which specific affective, behavioral, and cognitive facets are associated with distinct perceived outcomes. The model offers a theoretically structured basis for further research on responsible GenAI engagement, while its cross-sectional self-report design precludes causal or objective-performance conclusions.

## 1. Introduction

Generative artificial intelligence (GenAI) is becoming integrated into how university students generate ideas, draft and refine content, evaluate information, and collaborate with others (McDonald et al., 2025). Unlike conventional educational technologies that primarily provide access to information or structured learning activities, GenAI systems produce new content in response to users’ instructions and participate iteratively in academic tasks (Jwair, 2025). Meaningful use of GenAI, therefore, requires more than adoption or frequent use. Students must decide how to formulate prompts, evaluate generated information, revise outputs, disclose AI assistance, and determine when AI responses should be trusted or questioned (Dwivedi et al., 2023; Long & Magerko, 2020; Shneiderman, 2020). Understanding these processes is particularly important in higher education, where the potential benefits of GenAI coexist with concerns about inaccuracy, overreliance, authorship, academic integrity, and poorly calibrated trust.

Technology-acceptance frameworks provide important explanations of why individuals intend to adopt and use technologies. The Technology Acceptance Model emphasizes perceived usefulness and perceived ease of use (Davis, 1989), whereas the Unified Theory of Acceptance and Use of Technology incorporates performance expectancy, effort expectancy, social influence, and facilitating conditions (Venkatesh et al., 2012). These frameworks are valuable for explaining technology acceptance and intended use, but they do not fully capture what users think, feel, and do after they begin working with a generative system. Two students may use the same GenAI tool with similar frequency while engaging with it in fundamentally different ways: one may critically verify and refine its responses, whereas another may accept its output without evaluation. An engagement perspective can therefore complement acceptance-oriented frameworks by examining the quality and form of users’ involvement during human–AI interaction.

Drawing on Astin’s (1999) conception of involvement as the physical and psychological energy devoted to an educational experience and the multidimensional engagement framework developed by Fredricks et al. (2004), the present study conceptualizes AI engagement as the affective, behavioral, and cognitive investment that users direct toward their interactions with GenAI. Affective engagement concerns the emotions experienced during AI use; behavioral engagement concerns the actions through which users generate, check, revise, discuss, and acknowledge AI-supported work; and cognitive engagement concerns the planning, monitoring, evaluation, and ethical reasoning applied during these interactions. This perspective distinguishes engagement from usage frequency, satisfaction, and intention to adopt because it concerns how users participate in AI-assisted activities after adoption.

The conventional Affective–Behavioral–Cognitive model may nevertheless be too broad to capture theoretically important differences within each engagement domain. McChesney et al. (2025) proposed the ABC+ model of learning engagement, which differentiates affective engagement according to emotional valence and organizes behavioral and cognitive engagement according to the activity spaces in which engagement occurs. Their findings indicate that positive and negative affect should not necessarily be represented as opposite ends of a single continuum and that the behaviors and cognitive strategies required for engagement may differ across activity contexts. This context-sensitive logic is particularly relevant to GenAI because working with a generative system involves functionally different activities, including creating content, evaluating its reliability, and discussing or sharing AI-assisted work.

Extending this logic to GenAI-supported learning, the present study specifies seven engagement facets. The affective domain distinguishes Positive AI Affect, such as enjoyment, curiosity, and motivation, from Negative AI Affect, including anxiety, frustration, and discomfort. The behavioral domain differentiates Creation Behavior, Verification Behavior, and Community Behavior. Creation Behavior represents the iterative generation and refinement of ideas; Verification Behavior concerns fact-checking, source comparison, correction, and evaluation against academic and ethical standards; and Community Behavior captures discussion, sharing, feedback, peer learning, and acknowledgment practices surrounding AI-assisted work. The cognitive domain differentiates Strategic Cognition from Critical Cognition. Strategic Cognition concerns planning, task organization, tool and prompt selection, and adaptive regulation, whereas Critical Cognition concerns scrutiny of evidence, potential bias, ethical implications, and the reliability of AI-generated reasoning.

This extension is intended to provide more than a transfer of the ABC+ terminology from conventional learning environments to an AI context. Its conceptual contribution lies in distinguishing engagement configurations relevant to productive outcomes from those relevant to responsible and epistemically cautious AI use. Creation Behavior and Strategic Cognition concern how students organize and develop AI-assisted work, whereas Verification Behavior and Critical Cognition concern how they evaluate its accuracy, integrity, and trustworthiness. Community Behavior introduces a social dimension through which AI-supported ideas and norms of responsible use are discussed, evaluated, and acknowledged. Positive and Negative AI Affect may also exhibit different associations with these processes rather than functioning as interchangeable indicators of a single emotional orientation. This differentiation helps explain why frequent or enthusiastic use of GenAI alone may not correspond to responsible, creative, or appropriately calibrated engagement.

The present study examines four self-reported AI-supported outcomes: perceived Output Quality, Integrity, Creativity, and Trust Calibration. Perceived Output Quality concerns students’ assessments of the clarity, accuracy, effectiveness, and overall quality of their AI-supported work. Integrity concerns reported disclosure, citation, originality, honesty, and transparency practices. Creativity reflects students’ perceptions that AI helps them generate original or unconventional ideas. Trust Calibration concerns their reported ability to determine when AI outputs can be relied upon and when further verification is necessary. These constructs represent students’ perceptions and self-reported practices; they are not objective evaluations of the quality, creativity, integrity, or trustworthiness of independently assessed work products. This study examines these relationships among 1250 domestic and international students representing 15 nationalities and studying at eight universities in China. This context is theoretically relevant because domestic and international students may navigate different academic conventions, language demands, institutional expectations, and prior experiences with GenAI. Nevertheless, the evidence should be interpreted within this specific population and institutional setting rather than generalized to all higher-education students or national systems. Accordingly, the study addresses the following research questions:

1. How are the affective, behavioral, and cognitive facets of GenAI engagement associated with perceived Output Quality, Integrity, Creativity, and Trust Calibration among students enrolled at universities in China?
2. To what extent does the AI Engagement ABC+ Model explain and predict variation in these self-reported outcomes in this sample?

By examining differentiated engagement facets rather than representing AI engagement as a single score, this study seeks to clarify how specific ways of thinking, feeling, and acting with GenAI are associated with productive, creative, integrity-related, and trust-related outcomes. It thereby provides a context-sensitive examination of the ABC+ framework in GenAI-supported higher education while recognizing the cross-sectional, self-reported, and context-specific character of the evidence.

### 1.1. Background and Prior Studies

Engagement has commonly been conceptualized as a multidimensional construct comprising affective, behavioral, and cognitive investment in learning activities (Austin, 1962; Fredricks et al., 2004). Affective engagement encompasses emotional responses such as interest, enjoyment, anxiety, or frustration. Behavioral engagement concerns participation, persistence, effort, and task-directed action, whereas cognitive engagement encompasses strategic thinking, self-regulation, and deep processing. Although these dimensions are related, they represent distinguishable ways through which individuals become involved in educational activities.

Empirical research has generally supported the multidimensional character of engagement. Ben-Eliyahu et al. (2018) examined affective, behavioral, and cognitive engagement across formal and informal learning contexts and demonstrated that engagement varies according to both its dimension and context. Similarly, Miranda-Zapata et al. (2018) examined relationships among engagement dimensions and educational outcomes. Collectively, this literature indicates that representing engagement solely through participation frequency or an overall score can obscure meaningful differences in how individuals emotionally, behaviorally, and cognitively approach learning activities.

Technology-mediated learning creates additional reasons to treat engagement as multidimensional and context-sensitive. Digital environments can require learners to navigate information, regulate attention, evaluate sources, communicate with others, and adapt their strategies in response to system feedback. However, technology-acceptance frameworks such as TAM and UTAUT primarily explain acceptance, intention, and use rather than the quality of users’ affective, behavioral, and cognitive involvement during particular technology-supported tasks (Davis, 1989; Venkatesh et al., 2012). This distinction becomes especially important for GenAI because using the system does not ensure that its outputs are evaluated, refined, acknowledged, or used responsibly. McChesney et al. (2025) advanced this discussion through the ABC+ model of learning engagement. The model differentiates affective engagement according to positive and negative emotional valence and treats behavioral and cognitive engagement as sensitive to distinct activity spaces. In their educational application, the behaviors and cognitive strategies associated with lectures, recitations, examinations, and other learning activities were not assumed to be interchangeable. This refinement suggests that engagement should be understood in relation to what individuals are doing and what forms of thinking the activity requires.

GenAI-supported learning similarly comprises distinguishable activity contexts, but these contexts differ from those examined in conventional classroom engagement. Students may use GenAI to generate and refine ideas, verify claims and sources, or discuss and share AI-supported work with others (Rehman et al., 2026; Sarwar et al., 2026). These creation, verification, and community activities involve different behavioral demands. They also require different forms of cognitive regulation: strategic cognition supports planning and adaptive task completion, whereas critical cognition supports the evaluation of bias, evidence, ethical implications, and reliability. Applying the activity-space principle to these GenAI-specific practices therefore provides a theoretically grounded means of examining why different engagement facets may be associated with different perceived outcomes. The proposed model consequently does not assume that all forms of engagement should relate uniformly to all outcomes. Productive and creative outcomes may be more closely associated with positive affect, creation-oriented behavior, and strategic cognition, whereas integrity and calibrated trust may be more closely associated with verification-oriented behavior and critical cognition. Negative affect may also exhibit a selective rather than uniform relationship with responsible AI use. These differentiated expectations provide the basis for the hypotheses developed in the following theoretical framework.

### 1.2. Theoretical Framework

#### 1.2.1. From the ABC Model to AI Engagement

Engagement has long been regarded as important for understanding motivation, learning, and performance across educational activities. Foundational scholarship conceptualizes engagement in terms of the physical and psychological energy individuals invest in an experience and distinguishes affective, behavioral, and cognitive forms of involvement (Astin, 1999; Fredricks et al., 2004). Affective engagement encompasses emotional responses such as interest, enjoyment, anxiety, and frustration; behavioral engagement concerns participation, effort, persistence, and task-directed action; and cognitive engagement involves strategic thinking, self-regulation, reflection, and deep processing. Although related, these dimensions represent distinguishable forms of involvement that can exhibit different relationships with learning processes and outcomes (Ben-Eliyahu et al., 2018; Miranda-Zapata et al., 2018). Generative artificial intelligence introduces an engagement context that differs from conventional technology use. Students do not merely access information through GenAI; they formulate instructions, interpret newly generated content, revise outputs, evaluate claims, and decide whether and how to use AI as assistance. Consequently, adoption of a GenAI system does not guarantee careful or responsible engagement with its outputs. Two students may report similar levels of AI use while differing substantially in emotional orientation, prompting and revision practices, verification behavior, and critical scrutiny. This distinction between the amount of AI use and the quality of engagement during AI use is central to the present framework.

Technology-adoption frameworks such as the Technology Acceptance Model and the Unified Theory of Acceptance and Use of Technology explain important antecedents of technology acceptance and intended use, including perceived usefulness, ease of use, performance expectancy, and facilitating conditions (Davis, 1989; Venkatesh et al., 2012). However, these frameworks were not principally designed to differentiate the affective, behavioral, and cognitive investment that occurs during continuing interaction with a generative system. Engagement, therefore, complements technology-acceptance perspectives by focusing on what students think, feel, and do while using GenAI, rather than solely on whether they intend to adopt it (Dwivedi et al., 2023; Jarrahi et al., 2025). Emerging research similarly suggests that human–AI interaction can involve different emotional orientations, ranging from curiosity and enthusiasm to anxiety and distrust; different behavioral practices, from exploratory generation to deliberate verification; and different levels of cognitive investment, from strategic reasoning to superficial reliance on automated outputs (Lee et al., 2023; Zhang & Dafoe, 2019; Long & Magerko, 2020). These differences indicate that AI engagement should not be captured solely by usage frequency, satisfaction, or a single overall score. Instead, AI engagement is conceptualized here as a context-sensitive configuration of affective, behavioral, and cognitive involvement that occurs during students’ continuing interaction with GenAI-generated content.

#### 1.2.2. Extending ABC+ to GenAI-Supported Learning

McChesney et al. (2025) refined the conventional ABC framework by differentiating positive and negative affect and by demonstrating that behavioral and cognitive engagement can vary across activity spaces. In their model, activity spaces refer to contexts with different goals and demands, such as lectures, recitations, and examinations. This refinement suggests that engagement should be analyzed in relation to the type of activity being performed rather than assumed to operate uniformly across contexts.

The present framework adopts the context-sensitivity principle of ABC+ rather than reproducing the original activity-space structure unchanged. In McChesney et al.’s (2025) model, activity spaces were defined by conventional educational settings. The present study does not assume that these classroom-based activity spaces transfer directly to human–AI interaction. Instead, it develops a domain-specific adaptation based on recurring functions of GenAI-supported activity. Behavioral engagement is differentiated according to creation, verification, and community participation, whereas cognitive engagement is differentiated according to strategic and critical regulatory functions. Accordingly, the proposed model should be understood as a domain-specific theoretical adaptation of ABC+, not as a direct replication of its original measurement architecture.

This adaptation changes the analytical unit from engagement across physical or instructional settings to engagement with the functions and demands of GenAI-supported activity. The behavioral domain therefore differentiates Creation Behavior, Verification Behavior, and Community Behavior. Creation Behavior includes brainstorming, prompt experimentation, iterative refinement, exploration of alternatives, and integration of AI-generated content with students’ own ideas. Verification Behavior includes fact-checking, comparison with reliable sources, correction of inaccuracies, and evaluation against ethical or academic standards. Community Behavior concerns sharing AI-related knowledge, discussing generated ideas, learning from peers, providing feedback, and acknowledging AI assistance. These behavioral facets represent substantively different ways of acting with and around AI-generated content rather than interchangeable indicators of general participation.

The cognitive domain differentiates Strategic Cognition and Critical Cognition according to their primary regulatory functions. Strategic Cognition concerns planning how to use AI, selecting suitable tools or prompts, organizing tasks, monitoring progress, and adapting one’s approach. Critical Cognition concerns evaluating evidence and sources, questioning possible bias, considering ethical implications, and scrutinizing how AI-generated conclusions are reached. These processes are theoretically related but not interchangeable: Strategic Cognition principally regulates how an AI-supported task is accomplished, whereas Critical Cognition regulates whether the information and practices involved are credible, ethically defensible, and worthy of trust (Lee et al., 2023; Long & Magerko, 2020; Zhu et al., 2025). The distinction therefore separates task-oriented regulation from epistemic and ethical regulation, providing a theoretical basis for expecting the two cognitive facets to be associated with different types of outcomes. The affective domain differentiates Positive AI Affect from Negative AI Affect. Positive AI Affect captures enjoyment, curiosity, motivation, excitement, and flow during GenAI use. Negative AI Affect captures anxiety about dependence, frustration with inadequate responses, unease about AI decision-making, concern about mistakes, and discomfort with trusting generated information. Treating these orientations separately recognizes that the absence of positive emotion is not necessarily equivalent to the presence of anxiety or distrust. Positive and negative affect may therefore exhibit different relationships with the outcomes of AI-supported activity (Fredricks et al., 2004; Lee et al., 2023; Long & Magerko, 2020; Marvi et al., 2025; Zhu et al., 2025). Taken together, these distinctions produce seven theoretically differentiated engagement facets: Positive AI Affect, Negative AI Affect, Creation Behavior, Verification Behavior, Community Behavior, Strategic Cognition, and Critical Cognition. The resulting AI Engagement ABC+ Model does not specify a higher-order engagement construct, statistical interactions among affect, behavior, and cognition, or a causal sequence linking the three domains. Instead, it proposes that the seven first-order facets exhibit differentiated associations with four self-reported AI-supported outcomes: perceived Output Quality, Creativity, Integrity, and Trust Calibration. The conceptual contribution therefore lies not simply in transferring the ABC labels to GenAI use, but in specifying how affective valence, behavioral functions, and cognitive regulatory functions become differentiated within GenAI-supported learning.

#### 1.2.3. Perceived AI-Supported Outcomes

Perceived Output Quality refers to students’ assessments that using AI helps them produce clear, accurate, effective, and higher-quality work. Creativity concerns students’ perceptions that AI supports original, innovative, or unconventional thinking. Integrity reflects self-reported practices of disclosure, appropriate citation, originality, honesty, and transparency. Trust Calibration represents students’ reported ability to determine when AI outputs can reasonably be relied upon and when further scrutiny or verification is necessary. These outcomes represent perceptions and self-reported practices rather than independently evaluated task performance. Accordingly, the model examines associations between students’ reported forms of GenAI engagement and their perceived AI-supported outcomes; it does not test whether engagement causes changes in objectively assessed performance. This qualification is particularly important because the cross-sectional survey design cannot establish temporal or causal direction. The structural model follows a functional-alignment principle. Paths are specified when the defining content of an engagement facet corresponds theoretically to the demands represented by an outcome. Generative and task-regulatory facets are linked principally to perceived productive and creative outcomes; evaluative and epistemic-regulatory facets are linked principally to Integrity and Trust Calibration; and socially situated engagement is linked to outcomes involving idea exchange and accountable practice. The model therefore does not assume that every engagement facet should be associated with every outcome. This theoretically restricted specification provides a more parsimonious and falsifiable test than an exhaustive model connecting every engagement facet with every outcome.

#### 1.2.4. Affective Engagement and Perceived Outcomes

Positive affect can broaden attention, encourage exploration, and sustain willingness to experiment with alternative ideas (Fredricks et al., 2004). During GenAI-supported work, enjoyment, curiosity, motivation, and flow may encourage students to explore prompts, refine responses, and remain involved in the task. These processes provide a theoretical basis for expecting Positive AI Affect to be positively associated with both perceived Output Quality and Creativity. Positive emotion may support perceived Output Quality by sustaining iterative engagement and may support Creativity by increasing openness to alternative ideas and unconventional combinations (Fredricks et al., 2004; Marvi et al., 2025).

The structural model follows a functional-alignment principle. Paths were specified when the defining content of an engagement facet corresponded theoretically to the demands represented by an outcome. Generative and task-regulatory facets were linked principally to perceived productive and creative outcomes; evaluative and epistemic-regulatory facets were linked principally to integrity and calibrated trust; and socially situated engagement was linked to outcomes involving idea exchange and accountable practice. The model therefore does not assume that every engagement facet should predict every outcome. This theoretically restricted specification provides a more parsimonious and falsifiable test than an exhaustive all-to-all model.

**H1a.**  *Positive AI Affect is positively associated with perceived Output Quality.*

**H1b.**  *Positive AI Affect is positively associated with Creativity.*

Negative AI Affect represents anxiety, frustration, unease, concern about errors, and discomfort with trusting AI-generated information. Although some skepticism may motivate careful evaluation, the present measures do not distinguish moderate or constructive skepticism from excessive negative affect. The proposed relationships therefore concern the observed linear Negative AI Affect construct rather than an untested curvilinear pattern of “optimal” negativity. Persistent anxiety and discomfort may inhibit balanced trust judgments. They may also be negatively associated with self-reported Integrity if uncertainty, frustration, or avoidance interferes with the sustained attention and self-regulation required for careful disclosure, attribution, and responsible AI use. Nevertheless, the expected association with Integrity is theoretically less direct than the expected association with Trust Calibration because the Negative AI Affect indicators assess emotional responses rather than ethical conduct (Lee et al., 2023; Long & Magerko, 2020; Zhu et al., 2025).

**H2a.**  *Negative AI Affect is negatively associated with Integrity.*

**H2b.**  *Negative AI Affect is negatively associated with Trust Calibration.*

#### 1.2.5. Behavioral Engagement and Perceived Outcomes

Creation Behavior reflects active and iterative collaboration with GenAI through brainstorming, prompt experimentation, output refinement, exploration of alternatives, and integration of AI-generated material with personal ideas. These activities can help users elaborate on their goals, compare possible responses, and progressively align generated content with task requirements. They may consequently be associated with stronger perceptions of both Output Quality and Creativity. This expectation is consistent with accounts of AI-supported creativity as involving active generation, comparison, iteration, and refinement rather than the passive receipt of generated ideas (Nandagopal, 2025; Pena et al., 2026; Wei et al., 2025).

**H3a.**  *Creation Behavior is positively associated with perceived Output Quality.*

**H3b.**  *Creation Behavior is positively associated with Creativity.*

Verification Behavior concerns deliberate scrutiny of AI-generated information. Fact-checking, consulting reliable sources, correcting inaccuracies, examining ethical or academic compliance, and evaluating material before submission can reduce uncritical reliance on generated content. These practices should be associated with perceived Output Quality because they facilitate correction and refinement; with Integrity because they support accuracy, attribution, and compliance; and with Trust Calibration because they provide evidence for deciding when reliance on AI is appropriate (Jarrahi et al., 2025; Zhu et al., 2025).

**H4a.**  *Verification Behavior is positively associated with perceived Output Quality.*

**H4b.**  *Verification Behavior is positively associated with Integrity.*

**H4c.**  *Verification Behavior is positively associated with Trust Calibration.*

Community Behavior introduces the social dimension of GenAI engagement. Discussing AI-generated ideas, sharing useful prompts or insights, learning from peers, offering feedback, and acknowledging AI assistance can expose students to alternative perspectives and socially negotiated standards of responsible use. Peer exchange may support Creativity by encouraging recombination and elaboration of ideas. It may also support Integrity by normalizing acknowledgment, feedback, transparency, and shared accountability surrounding AI-supported work (Juan et al., 2026).

**H5a.**  *Community Behavior is positively associated with Creativity.*

**H5b.**  *Community Behavior is positively associated with Integrity.*

#### 1.2.6. Cognitive Engagement and Perceived Outcomes

Strategic Cognition concerns goal setting, task planning, tool and prompt selection, organization, monitoring, and adaptation. These self-regulatory processes can help students align AI interaction with task objectives and revise their approach when initial responses are inadequate. Strategic Cognition is therefore expected to be associated with perceived Output Quality through goal alignment and adaptive task management and with Creativity through purposeful exploration of tools, prompts, and alternative solutions (Choudhuri et al., 2025; Hao et al., 2026; Rai et al., 2026).

**H6a.**  *Strategic Cognition is positively associated with perceived Output Quality.*

**H6b.**  *Strategic Cognition is positively associated with Creativity.*

Critical Cognition concerns questioning bias, evaluating sources, examining ethical implications, and scrutinizing the reasoning represented in AI-generated responses. These practices can strengthen Integrity by encouraging responsible attribution, ethical reflection, and resistance to misrepresentation. They can also support Trust Calibration by helping users distinguish credible outputs from those requiring further verification. Critical cognition thus provides an epistemic and ethical form of regulation that is theoretically distinct from the task-management function of strategic cognition (Rai et al., 2026).

**H7a.**  *Critical Cognition is positively associated with Integrity.*

**H7b.**  *Critical Cognition is positively associated with Trust Calibration.*

#### 1.2.7. Theoretical Contribution of the AI Engagement ABC+ Model

The proposed framework makes three related theoretical contributions. First, it shifts analytical attention from GenAI acceptance to the quality and form of students’ involvement after adoption. Technology-acceptance perspectives explain whether individuals are inclined to adopt or use a system, whereas the AI Engagement ABC+ Model differentiates how they participate affectively, behaviorally, and cognitively during AI-supported activity (Isaeva et al., 2026; Luo & Yin, 2026). The framework therefore complements rather than replaces established acceptance models. Its theoretical focus is post-adoption engagement: how students regulate their emotional, behavioral, and cognitive involvement once interaction with GenAI has begun.

Second, the framework develops a domain-specific adaptation of the context-sensitivity principle underlying ABC+. GenAI-supported learning involves recurring functions that are not captured adequately by an undifferentiated measure of behavioral engagement. Students may generate and refine content, scrutinize the accuracy and acceptability of that content, and discuss or acknowledge AI-assisted work within a social community. Differentiating Creation, Verification, and Community Behavior therefore identifies how behavioral engagement changes according to the immediate function of GenAI-supported activity. This represents more than relabeling the behavioral component of ABC: it specifies functionally distinct forms of participation that are theoretically aligned with different perceived outcomes.

Third, the framework distinguishes productive regulation from epistemic and ethical regulation. Creation Behavior and Strategic Cognition principally concern developing, organizing, and adapting AI-supported work, whereas Verification Behavior and Critical Cognition principally concern evaluating its accuracy, integrity, limitations, and trustworthiness. Community Behavior connects these functions with socially situated processes of idea exchange, feedback, acknowledgment, and accountability. This functional differentiation provides a theoretical explanation for why the same engagement facet need not be equally relevant to perceived Output Quality, Creativity, Integrity, and Trust Calibration. It also explains why greater engagement cannot automatically be interpreted as better engagement: its implications depend on the function and quality of the involvement being enacted.

More specifically, the framework identifies three interpretive orientations within GenAI engagement. A generative orientation, represented principally by Positive AI Affect and Creation Behavior, supports exploration and idea development. A strategic orientation, represented by Strategic Cognition, regulates goal alignment, prompt selection, and adaptive task management. An evaluative-accountable orientation, represented by Verification Behavior, Critical Cognition, and aspects of Community Behavior, regulates evidentiary scrutiny, ethical judgment, transparency, and socially shared standards of responsible AI use. These orientations are not estimated as additional latent variables; rather, they clarify the distinct theoretical functions performed by the seven measured engagement facets. They should therefore be understood as an interpretive organization of the framework, not as second-order constructs or empirically estimated dimensions.

**Figure 1:**
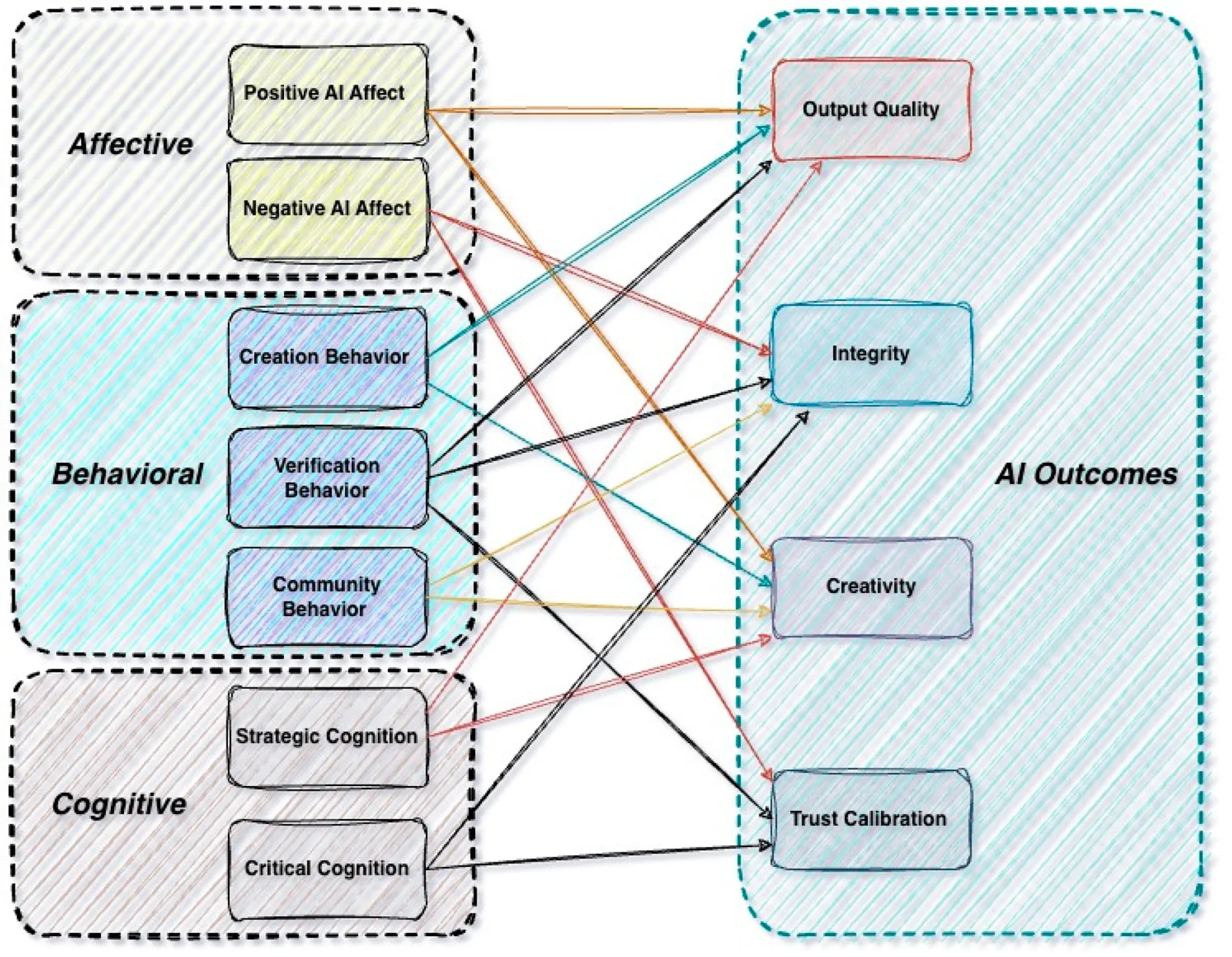
Conceptual Model of the AI Engagement ABC+ Framework.

The model is consequently falsifiable through a restricted set of theoretically aligned structural relationships rather than through the general proposition that more engagement uniformly produces better outcomes. Figure 1 presents the resulting framework, and its arrows represent the 15 directional associations derived in H1a–H7b. The model does not specify causal effects, statistical interactions, mediation, or a higher-order AI engagement construct. It evaluates whether seven first-order engagement facets show distinct associations with four self-reported AI-supported outcomes among Chinese domestic and international students at eight universities in China. Accordingly, support for the framework depends not merely on the statistical significance of individual paths but on whether the overall pattern of associations corresponds to its proposed functional differentiation.

## 2. Materials and Methods

This study employed a cross-sectional, explanatory quantitative design using a structured online questionnaire to test the proposed AI Engagement ABC+ Model. A cross-sectional design was appropriate given the study’s aim to examine the structural relationships among multiple engagement dimensions and self-reported AI-supported outcomes at a single point in time (Hunziker & Blankenagel, 2024). Given the cross-sectional design, the estimated structural relationships are interpreted as conditional associations rather than evidence of causal effects. Partial least squares structural equation modeling (PLS-SEM) was selected as the primary analytical approach because it permits the simultaneous assessment of a multi-construct measurement model and multiple structural relationships while also supporting the evaluation of explained variance and out-of-sample predictive performance (Hair et al., 2020). The model contained 11 first-order reflective constructs: seven GenAI engagement facets, Positive AI Affect, Negative AI Affect, Creation Behavior, Verification Behavior, Community Behavior, Strategic Cognition, and Critical Cognition, and four self-reported AI-supported outcomes, perceived Output Quality, Integrity, Creativity, and Trust Calibration. No higher-order engagement construct, mediation, or interaction effects were specified. The structural model examined 15 theoretically derived relationships among these constructs.

### 2.1. Data Collection and Sampling

Data were collected between February and May 2025 from students enrolled at eight universities in China using nonprobability convenience sampling. The target population included Chinese domestic and international students. The final analytical sample comprised 1250 students from 15 nationalities: 470 Chinese domestic students (37.6%) and 780 international students from 14 other nationalities (62.4%). Studying this group provides an opportunity to examine GenAI engagement among students with varied educational and cultural backgrounds within the participating institutions. Because participants were recruited through convenience sampling, the findings should be interpreted in relation to this sample rather than generalized to all students in China or to international students as a whole. A nonprobability convenience-sampling strategy was used. The survey invitation was distributed through online communication channels commonly used by students, including WeChat, QQ, and university student groups. These channels enabled the researchers to reach students from different educational levels, disciplinary backgrounds, and nationalities within the participating universities. The questionnaire was administered in English through SurveyMonkey. English was selected as a shared language for a multinational sample and is commonly used in the academic literature on generative AI. The survey invitation asked enrolled students to proceed only if they could understand and respond to the English-language questionnaire and had previously used GenAI for academic purposes; those who did not meet these conditions were asked not to participate. Some international students may have met English-language proficiency requirements as part of their university admission; however, participants’ test scores and English proficiency were not independently assessed in this study. Approximately 1500 questionnaires were submitted. Before analysis, submissions were screened for completeness. Responses were excluded if the participant did not complete the questionnaire or did not provide gender or disciplinary information needed for the sample description. Education level was treated differently: participants could select “prefer not to respond,” and undisclosed or unknown education levels were retained in that category. In total, approximately 250 submissions were excluded, leaving 1250 responses for analysis. Because recruitment occurred partly through online student groups and the exact number of eligible individuals who viewed the invitation could not be independently established, the number of completed questionnaires is not interpreted as a formal survey response rate. Two follow-up reminders were distributed via the same recruitment channels during the data collection period. The final analytical sample comprised 1250 students representing 15 nationalities. The largest international group consisted of Pakistani students (*n* = 320, 25.6% of the total sample), followed by Yemeni students (*n* = 60, 4.8%) and Egyptian students (*n* = 55, 4.4%). Complete nationality information is reported in Appendix A. The sample included 655 male participants (52.4%) and 595 female participants (47.6%). Regarding educational level, 550 participants were undergraduates (44.0%), 350 were graduate students (28.0%), and 132 were doctoral students (10.6%). Educational-level information was not disclosed or was recorded as unknown for 218 participants (17.4%). The disciplinary composition included 764 students from STEM fields (61.1%) and 486 from non-STEM fields (38.9%). Thus, the sample included both STEM and non-STEM students, although STEM students constituted the larger group. Table 1 presents the principal demographic characteristics, while the complete distribution by nationality is reported in Appendix A. The sample provides evidence concerning a multinational group of students enrolled at the eight participating universities in China. However, the nonprobability sampling strategy does not support statistical generalization to all university students in China, all international students, or students in other higher-education systems.

### 2.2. Measurements

The questionnaire was developed to operationalize the AI Engagement ABC+ Model. The final instrument contained 51 items measuring 11 first-order constructs: seven GenAI engagement facets and four self-reported AI-supported outcomes. The engagement facets comprised Positive AI Affect (PAA; five items), Negative AI Affect (NAA; five items), Creation Behavior (CB; five items), Verification Behavior (VB; five items), Community Behavior (COM; five items), Strategic Cognition (SC; five items), and Critical Cognition (CC; five items). The outcome constructs comprised perceived Output Quality (OQ; four items), Integrity (IN; four items), Creativity (CR; four items), and Trust Calibration (TC; four items). The instrument-development process was guided by established recommendations for construct conceptualization, item generation, content review, and preliminary testing (DeVellis, 2003; Hinkin, 1998) and informed by established frameworks from engagement and human–AI interaction literature. The Affective–Behavioral–Cognitive (ABC) model (Fredricks et al., 2004) provided the structural foundation, while the ABC+ model (McChesney et al., 2025) informed the adaptation of engagement to the GenAI context. These sources informed the conceptual content of the item pool; the final wording was adapted to students’ continuing interactions with GenAI. Items were drafted to reflect the definition of each proposed construct and reviewed for relevance, clarity, and overlap before pilot testing. The items were context-specific adaptations informed by the cited literature rather than a direct reproduction of an existing validated GenAI engagement scale.

Rather than reproducing the original ABC+ activity-space architecture, the questionnaire adapted its context-sensitivity principle to recurring functions of GenAI-supported activity. Item development was also theoretically informed by scholarship on creativity (Amabile & Pratt, 2016), human–computer interaction (Shneiderman, 2020; Lee et al., 2023), and AI engagement (Dwivedi et al., 2023; Long & Magerko, 2020). These sources informed the conceptual content of the item pool; the final wording was adapted to the context of students’ continuing interaction with GenAI. The affective domain was represented by two distinct constructs rather than a single bipolar measure. Positive AI Affect captured enjoyment, curiosity, motivation, excitement, and flow during GenAI use. An example item was, “I feel motivated to use AI to improve my performance.” Negative AI Affect captured anxiety about dependence, frustration with inadequate responses, unease about AI decision-making, concern about mistakes, and discomfort with trusting generated information. An example item was, “I feel uncomfortable trusting AI-generated information.” Positive and negative affect were modeled separately because the absence of positive affect does not necessarily indicate the presence of anxiety, frustration, or distrust. The behavioral domain comprised three functionally differentiated constructs. Creation Behavior assessed brainstorming, prompt experimentation, iterative refinement, exploration of alternative solutions, and integration of AI-generated material with students’ own ideas. An example item was, “I experiment with different prompts to see what AI creates.” Verification Behavior assessed fact-checking, comparison with reliable sources, correction of inaccuracies, and evaluation of AI-generated content against ethical or academic standards. An example item was, “I fact-check AI-generated information before using it.” Community Behavior assessed sharing AI-related knowledge, discussing generated ideas with peers, learning from others, providing feedback, and acknowledging AI assistance. An example item was, “I discuss AI-generated ideas with my peers.” Creation, Verification, and Community Behavior were treated as distinct behavioral facets rather than as interchangeable indicators of a general behavioral-engagement construct.

The cognitive domain comprised Strategic Cognition and Critical Cognition. Strategic Cognition assessed planning, task organization, tool and prompt selection, monitoring, and adaptation during GenAI-supported activity. An example item was, “I plan how to use AI to reach specific goals.” Critical Cognition assessed evaluation of evidence and sources, questioning of potential bias, consideration of ethical implications, and scrutiny of AI-generated reasoning. An example item was, “I question whether AI-generated results are fair or biased.” The two constructs distinguish task-oriented regulation from epistemic and ethical regulation: Strategic Cognition concerns how students organize and adapt AI-supported activity, whereas Critical Cognition concerns whether generated information and associated practices are credible, defensible, and worthy of trust. Four constructs represented the self-reported AI-supported outcomes. Perceived Output Quality assessed the extent to which students believed AI helped them produce clear, accurate, effective, and higher-quality work; an example item was, “AI helps me produce higher-quality work.” Integrity assessed self-reported practices of disclosure, appropriate citation, originality, honesty, and transparency; an example item was, “I use AI in ways that uphold honesty and transparency.” Creativity assessed students’ perceptions that AI supported original, innovative, and unconventional thinking; an example item was, “AI helps me generate more original ideas.” Trust Calibration assessed students’ reported ability to determine when AI outputs could reasonably be relied upon and when further verification was required. An example item was “I know when to rely on AI and when to verify its results.” Accordingly, these measures represent perceived outcomes and self-reported practices rather than independently evaluated AI outputs or objectively assessed performance. All 51 items were rated on a five-point Likert-type scale ranging from 1 (strongly disagree) to 5 (strongly agree). These four outcomes were assessed through students’ self-reports: Output Quality and Creativity reflect students’ perceptions of their AI-supported work, Integrity reflects their reported practices, and Trust Calibration reflects their perceived ability to judge when AI outputs require verification. None was independently assessed through task outputs or observed behavior.

All 11 constructs were specified as first-order reflective measurement models. Within each construct, the items were treated as observable expressions of a common underlying reported orientation, practice, or perception and were therefore expected to covary. The measurement models were evaluated using indicator loadings, construct reliability, convergent validity, and discriminant validity. The complete item wording and standardized outer loadings are reported in the measurement-model results.

### 2.3. Expert Review and Pilot Testing

The initial item pool was reviewed by five academic experts with relevant expertise in educational psychology, AI ethics, and measurement design. The experts evaluated the items for conceptual relevance, clarity, construct alignment, potential overlap, and cross-cultural appropriateness. Their review was used to assess whether the item content adequately represented the proposed construct definitions and whether distinctions among closely related constructs, particularly Creation and Verification Behavior, Strategic and Critical Cognition, and Positive and Negative AI Affect, were sufficiently clear. The revised questionnaire was subsequently piloted with 50 students from two universities in China. Pilot participants provided feedback concerning item clarity, readability, interpretability, and completion burden. Their feedback resulted in minor linguistic refinements intended to improve comprehension without changing the conceptual definitions of the constructs. Following expert review and pilot testing, the finalized English-language questionnaire comprised 51 items measuring the 11 constructs described above. The questionnaire was then administered online through SurveyMonkey. Figure 1 presents the hypothesized structural relationships among the seven engagement facets and four self-reported AI-supported outcomes.

## 3. Results

Preliminary descriptive analyses were conducted in Stata 17 to examine data quality and distributional characteristics before model estimation. Although PLS-SEM does not require multivariate normality, the distributions of the study variables were examined as part of the preliminary screening process. Skewness and kurtosis values were within the prespecified limits (|skewness| < 2; |kurtosis| < 8) (Hair et al., 2019), indicating no substantial departures from univariate normality. Missingness was also examined because appropriate missing-data assessment is important in multivariate analysis (Enders, 2022). Descriptive statistics and internal consistency estimates for the 11 study constructs are presented in Table 2. Cronbach’s α values ranged from 0.811 to 0.901, whereas Dijkstra–Henseler’s rho_A values ranged from 0.815 to 0.903. All values exceeded the recommended threshold of 0.70, indicating satisfactory internal consistency. Construct means ranged from 2.82 for Negative AI Affect to 3.32 for Verification Behavior, while standard deviations ranged from 1.09 to 1.20. These results indicate that construct means were generally near or above the scale midpoint, with Negative AI Affect exhibiting the lowest mean and Verification Behavior the highest.

Pairwise correlations among the study constructs are presented in Table 3. The correlations were generally small to moderate, ranging in absolute magnitude from approximately zero to 0.38. Positive AI Affect and Negative AI Affect were negatively correlated (r = −0.22, *p* < .001), supporting their treatment as related but distinct affective orientations. Positive AI Affect was positively correlated with Output Quality (r = 0.28, *p* < .001) and Creativity (r = 0.31, *p* < .001), whereas Negative AI Affect was negatively correlated with all four outcomes, including Output Quality (r = −0.19, *p* < .001), Integrity (r = −0.16, *p* < .001), Creativity (r = −0.20, *p* < .001), and Trust Calibration (r = −0.22, *p* < .001). The behavioral constructs also exhibited differentiated associations with the outcomes. Creation Behavior was positively correlated with Output Quality (r = 0.29, *p* < .001) and Creativity (r = 0.38, *p* < .001), whereas Verification Behavior was positively correlated with Output Quality (r = 0.33, *p* < .001), Integrity (r = 0.35, *p* < .001), and Trust Calibration (r = 0.27, *p* < .001). Community Behavior was positively correlated with Integrity (r = 0.30, *p* < .001) and Creativity (r = 0.32, *p* < .001). Within the cognitive domain, Strategic Cognition was positively correlated with Output Quality (r = 0.24, *p* < .001) and Creativity (r = 0.25, *p* < .001), while Critical Cognition was positively correlated with Integrity (r = 0.28, *p* < .001) and Trust Calibration (r = 0.34, *p* < .001). These bivariate patterns were consistent with the differentiated structure proposed in the ABC+ Model. However, discriminant validity was evaluated separately using the HTMT and Fornell–Larcker results reported in Table 4. Before examining the structural relationships, the reflective measurement model was assessed for indicator reliability, internal consistency reliability, convergent validity, and discriminant validity. As reported in Table 5, all standardized outer loadings were statistically significant at *p* < .001 and ranged from 0.760 to 0.878, exceeding the recommended threshold of 0.70. Composite reliability values ranged from 0.876 to 0.927, while average variance extracted values ranged from 0.618 to 0.718. Because all composite reliability estimates exceeded 0.70 and all AVE values exceeded 0.50, the findings supported internal consistency reliability and convergent validity (Fornell & Larcker, 1981).

Discriminant validity was assessed using the heterotrait–monotrait ratio and the Fornell–Larcker criterion. As shown in Table 4A, HTMT estimates ranged from 0.021 to 0.430 and were therefore substantially below the conservative threshold of 0.85 (Henseler et al., 2015). Bootstrapped HTMT inference provided additional support: none of the 95% confidence intervals included 1.00, and the largest upper confidence limit was only 0.483. Inspection of the item cross-loadings showed that every indicator loaded more strongly on its assigned construct than on any other construct. For potentially overlapping items, COM5 loaded 0.840 on Community Behavior and 0.266 on Integrity; CC5 loaded 0.760 on Critical Cognition and 0.162 on Verification Behavior; and TC1 loaded 0.863 on Trust Calibration and 0.238 on Verification Behavior. These results support empirical separation of the specified constructs in this sample, while not ruling out overlap in the content of individual items.

As shown in Table 4B, the square root of the AVE for each construct also exceeded the absolute value of its correlations with every other construct. These findings collectively demonstrate that the 11 constructs possessed adequate indicator reliability, internal consistency reliability, convergent validity, and discriminant validity. The specified structural relationships were subsequently examined using Partial Least Squares Structural Equation Modeling in SmartPLS 4.1.1.1. Bootstrapping with 5000 resamples was used to estimate standard errors, t statistics, *p* values, and 95% confidence intervals for the structural relationships (Hair et al., 2019). Structural collinearity was assessed using inner-model variance inflation factors. Values ranged from 1.053 to 1.192, remaining substantially below conventional thresholds and indicating that predictor collinearity did not distort the estimated structural relationships.

The explanatory power of the structural model was assessed using R^2^ and adjusted R^2^ for each endogenous construct (Table 6). The model explained 25.7% of the variance in Creativity, 25.2% in Integrity, 20.8% in Output Quality, and 17.6% in Trust Calibration. These results indicate modest but meaningful explanatory power across the four self-reported AI-supported outcomes. Standardized path coefficients were interpreted alongside their bootstrapped standard errors, confidence intervals, and significance tests. Although coefficient magnitudes were considered descriptively in relation to the benchmarks proposed by Mayhew et al. (2016), their substantive interpretation was based primarily on theoretical relevance, statistical uncertainty, and relative magnitude within each outcome equation. The model’s out-of-sample predictive performance was assessed using PLSpredict and the cross-validated predictive ability test. All construct-level Q^2^_predict values were positive, ranging from 0.171 for Trust Calibration to 0.251 for Creativity, indicating predictive relevance for all endogenous constructs. At the indicator level, the PLS-SEM model produced lower RMSE values than the linear-model benchmark for all Creativity indicators, three of four Output Quality indicators, and three of four Trust Calibration indicators. Its relative predictive performance for Integrity was weaker, as it produced a lower RMSE for only one of the four indicators. CVPAT provided additional evidence of predictive ability: prediction loss was significantly lower than the indicator-average benchmark for every outcome and for the model overall. Thus, the model demonstrated meaningful out-of-sample predictive ability, although its performance relative to the linear benchmark varied across outcomes.

### 3.1. Structural-Model Results

**Figure 2:**
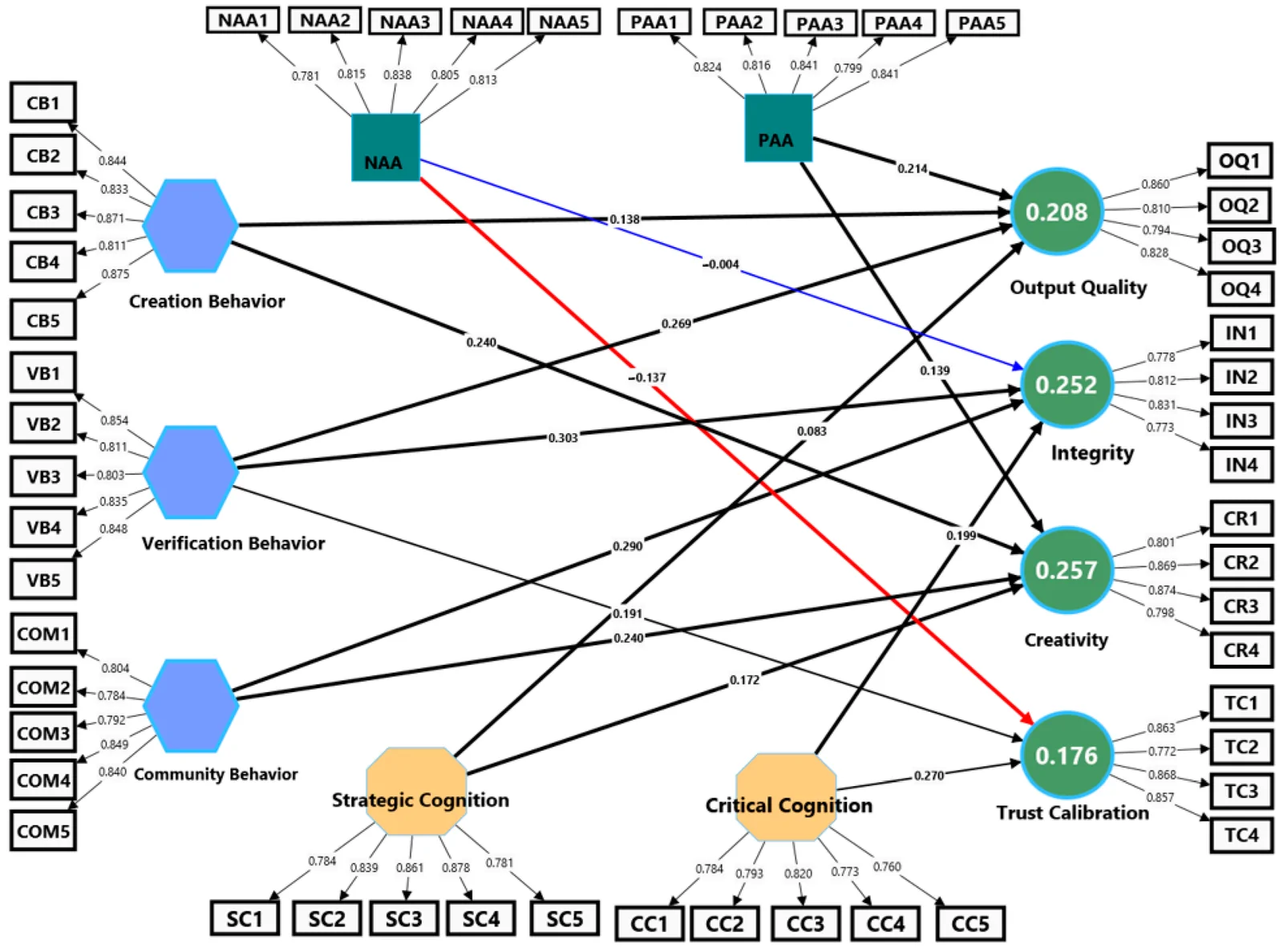
Structural Model Results for the AI Engagement ABC+ Framework. Note: TC = perceived trust calibration.

The structural-model results are presented in Figure 2 and summarized in Table 7. The AI Engagement ABC+ Model revealed differentiated relationships between the affective, behavioral, and cognitive facets of AI engagement and the four self-reported AI-supported outcomes. The model explained 25.7% of the variance in Creativity, 25.2% in Integrity, 20.8% in Output Quality, and 17.6% in Trust Calibration (Table 6). These values indicate modest but meaningful explanatory power across the four outcomes. Bootstrapping with 5000 resamples showed that 14 of the 15 tested structural relationships were statistically significant, with 95% confidence intervals that excluded zero. The only nonsignificant relationship was between Negative AI Affect and Integrity.

Addressing the first research question, the results showed that the affective, behavioral, and cognitive facets of AI engagement were associated with distinct combinations of self-reported AI-supported outcomes. Within the affective domain, Positive AI Affect was positively associated with Output Quality (β = 0.214, SE = 0.027, t = 8.018, *p* < .001, 95% CI [0.162, 0.267]) and Creativity (β = 0.139, SE = 0.027, t = 5.101, *p* < .001, 95% CI [0.085, 0.192]). The stronger coefficient for Output Quality suggests that students’ enjoyment, curiosity, motivation, and positive emotional involvement with AI were particularly relevant to their perceptions of producing accurate, coherent, and effective work. Positive affect was also associated with creativity, although this relationship was comparatively smaller. Negative AI Affect exhibited a more selective pattern. It was negatively associated with Trust Calibration (β = −0.137, SE = 0.026, t = 5.343, *p* < .001, 95% CI [−0.190, −0.089]), indicating that anxiety, frustration, discomfort, and concern about AI were associated with lower reported confidence in judging when AI should be relied upon or verified. By contrast, Negative AI Affect was not significantly associated with Integrity (β = −0.004, SE = 0.025, t = 0.174, *p* = .862, 95% CI [−0.055, 0.045]). The confidence interval for this relationship included zero, providing no evidence that negative emotional responses were related to students’ disclosure, citation, honesty, or transparency practices. Negative affect therefore appeared relevant to trust calibration rather than uniformly related to all responsible-AI outcomes. The behavioral dimensions also displayed differentiated relationships. Creation Behavior was positively associated with Creativity (β = 0.240, SE = 0.027, t = 8.888, *p* < .001, 95% CI [0.186, 0.292]) and Output Quality (β = 0.138, SE = 0.027, t = 5.035, *p* < .001, 95% CI [0.083, 0.191]). Thus, brainstorming with AI, experimenting with prompts, refining generated responses, exploring alternative solutions, and combining AI outputs with personal ideas were associated with both creative and higher-quality work. The stronger relationship with Creativity is consistent with the exploratory and generative character of these behaviors.

Community Behavior was positively associated with Creativity (β = 0.240, SE = 0.026, t = 9.357, *p* < .001, 95% CI [0.190, 0.291]) and Integrity (β = 0.290, SE = 0.024, t = 11.837, *p* < .001, 95% CI [0.242, 0.338]). Sharing prompts and insights, discussing AI-generated ideas with peers, learning from others, providing feedback, and acknowledging AI assistance were therefore associated with both creative performance and integrity-oriented practices. The comparatively strong association with Integrity suggests that responsible AI engagement may involve social practices of transparency, acknowledgment, peer learning, and shared accountability in addition to individual decision-making. Verification Behavior demonstrated the broadest pattern among the behavioral dimensions. It was positively associated with Integrity (β = 0.303, SE = 0.024, t = 12.657, *p* < .001, 95% CI [0.258, 0.351]), Output Quality (β = 0.269, SE = 0.026, t = 10.374, *p* < .001, 95% CI [0.220, 0.322]), and Trust Calibration (β = 0.191, SE = 0.026, t = 7.215, *p* < .001, 95% CI [0.139, 0.243]). Verification Behavior had the largest standardized coefficient among the predictors of both Integrity and Output Quality. These findings indicate that fact-checking AI-generated information, consulting reliable sources, correcting inaccuracies, assessing compliance with academic and ethical standards, and evaluating content before submission were particularly relevant to responsible and productive AI engagement. Within the cognitive domain, Strategic Cognition was positively associated with Creativity (β = 0.172, SE = 0.026, t = 6.536, *p* < .001, 95% CI [0.120, 0.224]) and Output Quality (β = 0.083, SE = 0.029, t = 2.882, *p* = .004, 95% CI [0.027, 0.139]). Planning how to use AI, selecting appropriate tools and prompts, organizing tasks, adjusting strategies, and reflecting on how AI could support performance were therefore associated with productive and creative outcomes. Although the Strategic Cognition → perceived Output Quality path was statistically significant (β = 0.083, 95% CI [0.027, 0.139], *p* = .004), the estimated association was small. Its statistical significance should not be interpreted as evidence of a substantial practical effect or objectively improved work. Critical Cognition was positively associated with Integrity (β = 0.199, SE = 0.026, t = 7.789, *p* < .001, 95% CI [0.149, 0.250]) and Perceived Trust Calibration (β = 0.270, SE = 0.025, t = 10.915, *p* < .001, 95% CI [0.223, 0.319]). Its coefficient for Perceived Trust Calibration was the largest among the predictors included in that outcome equation. Students who reported questioning potential bias, evaluating information sources, considering the ethical implications of AI-generated content, and critically examining AI reasoning also reported a higher perceived ability to judge when to rely on or verify AI and stronger integrity-related practices. These findings distinguish the roles of Critical Cognition and Strategic Cognition: Strategic Cognition was primarily associated with productive and creative outcomes, whereas Critical Cognition was associated with ethical judgment and calibrated trust.

An outcome-centered comparison further illustrates the differentiated structure of the model. Creativity was most strongly associated with Creation Behavior and Community Behavior, both of which had identical standardized coefficients of 0.240, followed by Strategic Cognition and Positive AI Affect. Output Quality was most strongly associated with Verification Behavior, followed by Positive AI Affect, Creation Behavior, and Strategic Cognition. Integrity was most strongly associated with Verification Behavior and Community Behavior, followed by Critical Cognition, whereas Negative AI Affect was unrelated to Integrity. Trust Calibration was most strongly associated with Critical Cognition, followed by Verification Behavior, while Negative AI Affect demonstrated a significant negative association. Thus, the largest estimated coefficient varied across the specified outcome equations. Instead, the findings support the ABC+ proposition that specific ways of feeling, acting, and thinking with AI are associated with different perceived outcomes and reported practices.

### 3.2. Explanatory and Out-of-Sample Predictive Performance

Addressing the second research question, the model exhibited modest explanatory power across the four self-reported AI-supported outcomes. The specified engagement facets explained 25.7% of the variance in Creativity, 25.2% in Integrity, 20.8% in perceived Output Quality, and 17.6% in Trust Calibration. Creativity therefore had the highest explained variance, followed closely by Integrity, whereas Trust Calibration had the lowest. These values indicate that the model accounted for a meaningful proportion of variance in the outcomes while leaving substantial variance attributable to factors not included in the framework. Out-of-sample predictive performance was assessed using PLSpredict and the cross-validated predictive ability test. At the construct level, all Q^2^_predict values were positive: 0.251 for Creativity, 0.246 for Integrity, 0.200 for perceived Output Quality, and 0.171 for Trust Calibration. At the indicator level, all 16 Q^2^_predict values were also positive, ranging from 0.077 to 0.217 (Appendix B). These findings indicate predictive relevance relative to the naïve mean-based benchmark for every endogenous construct and indicator.

CVPAT provided further evidence of predictive ability relative to the indicator-average benchmark. The PLS-SEM model produced significantly lower prediction loss for Creativity (loss difference = −0.345, (t = 10.056), (*p* < .001)), Integrity (loss difference = −0.307, (t = 9.686), (*p* < .001)), perceived Output Quality (loss difference = −0.268, (t = 8.759), (*p* < .001)), and Trust Calibration (loss difference = −0.240, (t = 7.989), (*p* < .001)). The overall loss difference was also negative and statistically significant (loss difference = −0.290, (t = 15.279), (*p* < .001)). The model therefore generated lower out-of-sample prediction error than the naïve indicator-average benchmark. Comparison with the linear-model benchmark produced less conclusive results. As reported in Appendix B, PLS-SEM generated lower RMSE values for all four Creativity indicators, three of the four perceived Output Quality indicators, and three of the four Trust Calibration indicators. For Integrity, it produced a lower RMSE for only one of the four indicators. Comparisons based on MAE were less favorable: PLS-SEM produced lower MAE for two Creativity indicators, no Integrity or perceived Output Quality indicators, and one Trust Calibration indicator; MAE was equal for one Trust Calibration indicator. Thus, relative performance varied according to both the indicator and the prediction-error measure.

The formal CVPAT comparison with the linear-model benchmark showed no statistically significant differences in prediction loss for Creativity (loss difference = −0.024, (t = 1.941), (*p* = .053)), Integrity (loss difference = 0.019, (t = 1.194), (*p* = .233)), perceived Output Quality (loss difference = −0.006, (t = 0.400), (*p* = .689)), Trust Calibration (loss difference = −0.009, (t = 0.537), (*p* = .591)), or the model overall (loss difference = −0.005, (t = 0.630), (*p* = .529)). Accordingly, the findings demonstrate out-of-sample predictive relevance and an advantage over the naïve indicator-average benchmark, but they do not establish a statistically significant predictive advantage over the linear-model benchmark. Taken together, the explanatory and predictive findings provide qualified support for the AI Engagement ABC+ Model as a differentiated framework for examining students’ self-reported engagement with GenAI. The model explained modest proportions of variance and demonstrated predictive relevance across all four outcomes. However, its predictive performance relative to the linear-model benchmark was mixed and statistically indistinguishable. These findings support cautious further investigation of the model rather than a conclusion that it possesses uniformly superior predictive performance (see Table 8A,B).

## 4. Discussion

This study examined how seven affective, behavioral, and cognitive facets of GenAI engagement were associated with four perceived AI-supported outcomes among students at eight universities in China. The facets showed different patterns of association with perceived Output Quality, Creativity, Integrity, and Trust Calibration, providing initial support for examining these forms of engagement separately.

Consistent with McChesney et al.’s (2025) extended ABC+ model, affective engagement was differentiated into Positive and Negative AI Affect, behavioral engagement into Creation, Verification, and Community Behavior, and cognitive engagement into Strategic and Critical Cognition. Fourteen of the 15 hypothesized relationships were supported. However, these facets were not modeled as interacting dimensions, components of a higher-order engagement construct, or causal determinants of the outcomes. Instead, the results showed that the seven first-order engagement facets exhibited differentiated conditional associations with four self-reported AI-supported outcomes: perceived Output Quality, Creativity, Integrity, and Trust Calibration. Creation Behavior and Strategic Cognition were principally associated with perceived productive and creative outcomes, whereas Verification Behavior and Critical Cognition were particularly relevant to Integrity and Trust Calibration. Community Behavior was associated with both Creativity and Integrity, thereby connecting idea exchange with socially situated practices of acknowledgement and accountability. The model explained 25.7% of the variance in Creativity, 25.2% in Integrity, 20.8% in perceived Output Quality, and 17.6% in Trust Calibration. These values indicate modest but meaningful explanatory power rather than strong explanatory validity. Moreover, the satisfactory reliability and validity results support the empirical distinguishability of the 11 measured constructs but do not, by themselves, confirm the complete theoretical framework.

### 4.1. Affective Engagement in AI Contexts

Affective engagement captures the emotional orientation individuals bring to their interactions with AI systems, an orientation that may encourage exploration or produce discomfort and resistance. Positive AI Affect was positively associated with perceived Output Quality (β = 0.214, *p* < .001) and Creativity (β = 0.139, *p* < .001). Thus, students who reported greater enjoyment, enthusiasm, curiosity, motivation, and flow during GenAI use also tended to report higher-quality AI-supported work and greater creativity. The stronger coefficient for perceived Output Quality suggests that Positive AI Affect was more closely associated with students’ assessments of producing clear and effective work than with their perceptions of originality.

This pattern can be situated within educational engagement research that connects positive emotional involvement with sustained participation in learning activities (Fredricks et al., 2004; Lee et al., 2023), and within research on emotional motivation in digital learning contexts (Rafiq-uz-Zaman et al., 2026). It is also consistent with the broaden-and-build account, which holds that positive emotions can broaden individuals’ momentary ranges of attention, thought, and possible action (Fredrickson, 2001). Experimental research has similarly shown that positive affect can facilitate creative problem-solving by supporting flexible associations and the combination of otherwise disconnected cognitive elements (Isen et al., 1987). Applied cautiously to the present findings, one plausible interpretation is that positive emotional involvement may help students remain engaged during repeated prompting, comparison, and refinement and may make them more willing to consider alternative ideas or combinations. Nevertheless, because engagement and outcomes were measured through a cross-sectional self-report questionnaire, the results cannot establish that positive affect caused improvements in objective Output Quality or Creativity. The reverse direction is also possible: students who perceive their AI-supported work as successful or creative may subsequently develop more positive emotional responses to GenAI.

Negative AI Affect demonstrated a more selective pattern. It was negatively associated with Trust Calibration (β = −0.137, *p* < .001), indicating that students who reported greater anxiety, frustration, unease, concern about errors, and discomfort with trusting AI also reported less confidence in their ability to determine when AI could appropriately be relied upon and when additional verification was necessary.

This relationship is best interpreted in light of the trust-calibration literature rather than as evidence that negative emotions inherently impair judgment. Trust calibration concerns the correspondence between a user’s level of trust and a system’s demonstrated capabilities, with both excessive and insufficient reliance representing potentially inappropriate responses (Liebherr et al., 2026). Research on trust in automation further indicates that appropriate reliance is shaped by interactions among users’ dispositions, situational experiences, and perceptions of system performance (Zeng et al., 2025). In AI-specific research, cognitive and emotional trust have likewise been shown to depend on factors such as perceived reliability, transparency, system representation, and user experience (Glikson & Woolley, 2020). This interpretation also aligns with human-centered AI scholarship emphasizing the importance of informed human oversight rather than either unquestioning reliance or wholesale rejection of AI systems (Henrique & Santos, 2024).

The present measure did not distinguish constructive skepticism from persistent anxiety, frustration, or distrust. The negative coefficient should therefore not be interpreted as evidence that all caution toward AI is undesirable. Constructive scrutiny may support appropriate reliance, whereas undifferentiated discomfort may coexist with uncertainty about when reliance or verification is warranted. The present linear, self-report measure cannot determine where that distinction occurs. In contrast, Negative AI Affect was not significantly associated with Integrity (β = −0.004, *p* = .862, 95% CI [−0.055, 0.045]); therefore, H2a was not supported. Although Negative AI Affect had a negative bivariate correlation with Integrity, this relationship was no longer evident after the other theoretically specified predictors of Integrity were considered simultaneously. This result suggests that students’ reported disclosure, citation, honesty, originality, and transparency practices were more directly associated with Verification Behavior, Community Behavior, and Critical Cognition than with their negative emotional responses to AI. The affective findings, therefore, do not support a general conclusion that positive emotions uniformly improve responsible AI use or that negative emotions uniformly undermine it. Nor did the study test whether an “optimal emotional balance” exists. Instead, the findings indicate outcome-specific associations: Positive AI Affect was related to perceived productive and creative outcomes, whereas Negative AI Affect was related to lower self-reported Trust Calibration but was unrelated to Integrity in the structural model. This differentiation supports treating positive and negative affect as distinct engagement facets rather than opposite endpoints of a single affective continuum.

### 4.2. Behavioral Engagement Across AI Activity Spaces

Behavioral engagement exhibited a differentiated pattern across the three GenAI-supported functions represented in the model: Creation Behavior, Verification Behavior, and Community Behavior. These constructs were not modeled as replications of the classroom activity spaces in the original ABC+ framework. Instead, they represent distinct behavioral functions through which students generate, evaluate, and socially discuss AI-supported content. Their different structural relationships support the proposition that behavioral engagement should not be represented as a single continuum of participation. Creation Behavior was positively associated with Creativity (β = 0.240, *p* < .001) and perceived Output Quality (β = 0.138, *p* < .001). Students who reported brainstorming with AI, experimenting with prompts, refining generated responses, considering alternative solutions, and combining AI-generated material with their own ideas also tended to report greater Creativity and higher Output Quality. The stronger coefficient for Creativity is consistent with the generative content of the construct, which emphasizes experimentation, iteration, and the recombination of ideas. This interpretation can be situated within self-regulated learning frameworks that emphasize planning, monitoring, reflection, and adaptation during learning activities (Green et al., 2020). Applied to GenAI-supported activity, prompt experimentation and iterative revision may provide opportunities for students to compare generated alternatives, assess progress toward a goal, and modify their approach. However, the present results do not demonstrate that these behaviors caused cognitive discovery, analytical reasoning, or creative fluency. They show that self-reported Creation Behavior was associated with students’ perceptions of Creativity and Output Quality. Verification Behavior demonstrated the broadest pattern among the behavioral facets. It was positively associated with Integrity (β = 0.303, *p* < .001), perceived Output Quality (β = 0.269, *p* < .001), and Trust Calibration (β = 0.191, *p* < .001). It also had the largest standardized coefficient among the predictors of both Integrity and perceived Output Quality. These findings indicate that students who reported fact-checking AI-generated information, consulting reliable sources, correcting inaccuracies, evaluating compliance with ethical or academic standards, and reviewing content before submission also reported stronger Integrity, higher Output Quality, and higher perceived ability to judge when to rely on or verify AI. Verification can be interpreted as a behavioral expression of critical AI literacy. AI-literacy frameworks identify the capacity to evaluate AI systems and their outputs critically as a central competency for effective interaction with AI (Long & Magerko, 2020). Guidance concerning GenAI in education similarly emphasizes human oversight, critical assessment of generated information, and the ethical validation of AI-supported practices (UNESCO, 2023). This interpretation extends Shneiderman (2020)’s call for human-centered AI by emphasizing that responsible use depends not only on system transparency and reliability but also on users’ willingness to examine generated content. Verification may therefore provide students with information relevant to deciding when reliance is warranted, which is consistent with the concept of appropriate reliance in the trust-calibration literature (Liebherr et al., 2026). Nevertheless, because the present measures were self-reported, the findings do not demonstrate that participants actually identified every inaccuracy or produced objectively more reliable work. Community Behavior was positively associated with Creativity (β = 0.240, *p* < .001) and Integrity (β = 0.290, *p* < .001). Students who reported sharing AI-related knowledge, discussing generated ideas, learning from peers, providing feedback, and acknowledging AI assistance also tended to report greater Creativity and stronger Integrity-related practices. The association with Creativity is consistent with the possibility that peer discussion exposes students to alternative perspectives and supports the elaboration or recombination of ideas. Its association with Integrity may reflect the role of acknowledgement, feedback, transparency, and socially shared expectations surrounding responsible AI use. This interpretation is consistent with sociocultural engagement perspectives that recognize interaction and participation with others as important components of engagement (Fredricks et al., 2004). It can also be interpreted cautiously through an activity-theoretical perspective, in which individual action is mediated by tools, community norms, and socially organized practices (Engeström, 2000). The current study did not directly observe peer interaction or measure the emergence of group norms. Consequently, it cannot establish that Community Behavior produced shared standards of credibility, fairness, or accountability. Rather, the results indicate that students’ reported social engagement surrounding AI use was associated with both perceived Creativity and self-reported Integrity. Taken together, the behavioral findings support functional differentiation among creating, verifying, and participating in an AI-related community. Creation Behavior was more closely associated with generative and productive outcomes; Verification Behavior was associated with productive, integrity-related, and trust-related outcomes; and Community Behavior was associated with creative and integrity-related outcomes. These results show that the three reported behavioral practices had different patterns of association with the perceived outcomes in the specified model.

### 4.3. Cognitive Engagement and Strategic Thinking

The cognitive findings support a distinction between Strategic Cognition and Critical Cognition. These constructs were modeled as related but empirically distinguishable regulatory functions, not as stages of a developmental continuum. Strategic Cognition concerns planning, goal setting, task organization, tool and prompt selection, monitoring, and adaptation. Critical Cognition concerns evaluating sources and evidence, questioning bias, considering ethical implications, and scrutinizing AI-generated reasoning. Strategic Cognition was positively associated with Creativity (β = 0.172, *p* < .001) and perceived Output Quality (β = 0.083, *p* = .004). Students who reported planning their use of AI, selecting tools and prompts deliberately, organizing their tasks, monitoring progress, and adapting their approach also tended to report greater Creativity and higher Output Quality. The association with perceived Output Quality was small (β = 0.083). Given the large sample, its statistically significant *p* value should be interpreted cautiously; the finding does not establish a substantial practical association or an improvement in objectively assessed output quality. This pattern can be interpreted through self-regulated learning theory, which emphasizes goal setting, strategic planning, monitoring, and adaptation as distinguishable processes through which learners regulate their activity (Greene et al., 2015). Recent studies in AI-supported learning environments (Knoth et al., 2024; Xu et al., 2025) similarly discuss the relevance of goal setting, strategic prompting, feedback evaluation, and metacognitive regulation. The present findings are consistent with these accounts but do not demonstrate that Strategic Cognition determined depth of understanding or objective performance. Instead, they indicate that students’ reported strategic regulation was positively associated with their perceptions of creative and higher-quality AI-supported work. Critical Cognition was positively associated with self-reported Integrity (β = 0.199, *p* < .001) and Perceived Trust Calibration (β = 0.270, *p* < .001). The latter was the largest estimated coefficient among the predictors included in the Perceived Trust Calibration equation. Students who reported questioning potential bias, evaluating sources, considering ethical implications, and scrutinizing AI-generated conclusions also reported more integrity-related practices and greater perceived ability to judge when AI outputs required verification.

This pattern is consistent with AI-literacy perspectives that identify critical evaluation, recognition of limitations, and ethical awareness as essential competencies for interacting responsibly with AI systems (Long & Magerko, 2020). It also aligns with human–AI complementarity perspectives emphasizing the continuing role of human judgment and oversight when algorithmic outputs are uncertain or potentially unreliable (Shneiderman, 2020; Dwivedi et al., 2023). The activity-theoretical perspective further provides a general basis for viewing cognition as mediated through interaction with cultural and technological tools (Engeström, 2000). However, the present model did not test whether Critical Cognition mediated relationships between affective and behavioral engagement, and it should not be described as an empirical “bridge” between those domains.

Recent human–AI collaboration research (Kong et al., 2025) also emphasizes the relevance of reflective evaluation when individuals interact with AI-generated information. The present results add to this discussion by showing that Critical Cognition was associated with self-reported Integrity and Trust Calibration within the specified PLS-SEM model. They do not establish that critical reflection eliminated overreliance or produced objectively more ethical behavior. Taken together, Strategic and Critical Cognition should be understood as complementary but distinct functions. Strategic Cognition regulates how students organize and adapt GenAI-supported tasks, whereas Critical Cognition regulates how they evaluate the credibility, limitations, and ethical acceptability of generated information. Their differentiated associations provide empirical support for separating task-oriented regulation from epistemic and ethical regulation: Strategic Cognition was associated with perceived Creativity and Output Quality, whereas Critical Cognition was associated with Integrity and Trust Calibration.

### 4.4. The AI Engagement ABC+ Framework: Integration and Theoretical Refinement

The findings provide qualified support for the AI Engagement ABC+ Model as a domain-specific framework for examining students’ engagement with GenAI. The framework complements technology-acceptance theories, which principally explain individuals’ intentions to adopt and use technology through constructs such as perceived usefulness, ease of use, performance expectancy, and facilitating conditions (Davis, 1989; Venkatesh et al., 2012). The present model does not empirically outperform or replace these theories because no formal comparison with rival models was conducted. Its distinct theoretical focus is post-adoption engagement: the forms of affective, behavioral, and cognitive involvement students report while interacting with GenAI-generated content.

The proposed model retains ABC+’s distinction among affective, behavioral, and cognitive engagement but adapts its context-sensitive approach to GenAI-supported academic work. Whereas McChesney et al. (2025) examined engagement across classroom activity spaces, the present model specifies behaviors involved in creating AI-supported content, verifying outputs, and discussing AI use with others. It also distinguishes strategic planning from critical evaluation during interaction with generated content. The observed outcome-specific associations offer initial evidence that these distinctions are useful for describing engagement in this sample. They do not establish that the proposed model fits or predicts better than the original ABC+ model or a technology-adoption model, because those alternatives were not estimated with these data.

The seven first-order engagement facets showed different patterns of association with the perceived outcomes in the specified model. However, the model does not position engagement as a “core determinant” of AI-supported outcomes, because its cross-sectional associations cannot establish causal determination and its outcomes were self-reported. Nor does it represent engagement as a higher-order energy system distributed across interacting channels. Instead, it distinguishes Positive and Negative AI Affect, Creation, Verification, and Community Behavior, and Strategic and Critical Cognition as related but empirically distinguishable facets with different conditional associations with perceived Output Quality, Creativity, Integrity, and Trust Calibration. The framework makes three related theoretical refinements. First, it differentiates emotional valence by treating Positive and Negative AI Affect as separate orientations rather than opposite endpoints of a single continuum. Their distinct structural relationships support this separation: Positive AI Affect was associated with perceived Output Quality and Creativity, whereas Negative AI Affect was associated with lower Trust Calibration but was unrelated to Integrity after accounting for the other predictors.

Second, the framework translates the context-sensitivity principle of ABC+ into three recurring behavioral functions of GenAI-supported activity: Creation, Verification, and Community Behavior. These are not direct replications of the classroom “activity spaces” in the original ABC+ framework. They identify different purposes served by students’ behavior with and around generated content: producing and refining ideas, examining the reliability and acceptability of outputs, and participating in socially situated exchanges concerning AI use. This distinction responds to the need for AI-engagement research to address critical evaluation, ethical responsibility, and human oversight in addition to technology acceptance and use (Dwivedi et al., 2023; UNESCO, 2023). Third, the framework distinguishes productive regulation from epistemic and ethical regulation. Strategic Cognition concerns planning, goal alignment, tool and prompt selection, monitoring, and adaptation, whereas Critical Cognition concerns evidentiary scrutiny, recognition of bias and limitations, ethical judgment, and calibrated reliance. This differentiation is consistent with AI-literacy perspectives that extend beyond operational use to include critical evaluation and responsible interaction (Long & Magerko, 2020). It also provides a theoretical explanation for the outcome-specific pattern observed in the structural model: Strategic Cognition was associated with perceived productive and creative outcomes, whereas Critical Cognition was associated with Integrity and Trust Calibration. The predictive assessment further qualifies the model’s explanatory contribution. Positive construct- and indicator-level Q2predict values, together with significantly lower CVPAT loss relative to the indicator-average benchmark, indicated out-of-sample predictive relevance beyond naïve prediction. However, performance against the linear-model benchmark varied across indicators and error measures, and CVPAT showed no statistically significant difference in prediction loss for any outcome or for the model overall. The findings therefore support the model’s predictive relevance but not a claim of superior predictive performance relative to a conventional linear benchmark. Its principal contribution lies in theoretically differentiating forms of GenAI engagement rather than demonstrating uniformly superior prediction.

The framework can be situated within broader socio-technical discussions of human–AI interaction (Long & Magerko, 2020; Zhang & Dafoe, 2019). It is also relevant to emerging discussions of hybrid intelligence, in which human judgment and algorithmic capabilities are combined during collaborative activity (Järvelä et al., 2025). However, the present study did not directly observe iterative human–AI feedback, compare human-only and AI-assisted performance, or test the co-evolution of human and algorithmic capabilities. Hybrid intelligence, therefore, provides a broader interpretive context rather than an empirically tested component of the model. The distinction between engagement and usage frequency is conceptual in this study. Frequency describes how often students use GenAI, whereas the proposed facets describe their reported emotional responses, practices, and thinking during that use. Because usage frequency was not included in the structural model, the study cannot determine whether the engagement facets explain additional variation beyond frequency or are more strongly associated with the outcomes. Accordingly, the AI Engagement ABC+ Model offers a theoretically differentiated and empirically testable framework for subsequent research on responsible GenAI engagement. Future studies should include both usage frequency and the engagement facets as predictors of the same outcomes to test their relative and incremental associations.

### 4.5. Limitation

This study has four principal limitations. First, nonprobability convenience sampling from eight universities in China limits generalizability beyond the participating institutions. Although the sample included Chinese and international students from 15 nationalities, recruitment through online student groups may have favored students who were more active online or interested in GenAI. Second, all predictors and outcomes were collected from the same respondents in a single questionnaire at one time, using the same five-point response format. This design may introduce common-method variance: general agreement tendencies, consistency in responding, or a desire to present one’s AI use favorably could contribute to the observed associations. This concern is especially relevant to self-reported Integrity, Verification Behavior, and Perceived Trust Calibration, which ask about practices or judgments that respondents may view as desirable. Although participation was anonymous, anonymity does not establish that these biases were absent. The estimated relationships should therefore be interpreted as associations among self-reports, rather than independent evidence that engagement practices improved outcomes. Future research should jointly measure ABC+ engagement facets and relevant TAM or UTAUT constructs to compare their explanatory and out-of-sample predictive performance against common outcomes and determine whether engagement provides an incremental contribution.

Future studies should combine questionnaires with behavioral records, independently rated work products, or measurements collected at different times. (Podsakoff et al., 2003). Third, the cross-sectional design does not establish temporal or causal direction. The reported paths represent conditional associations and cannot demonstrate that engagement facets produced changes in the outcomes. The English-language instrument represents an emerging, context-specific operationalization of AI engagement. Further research should cross-validate it across languages, cultures, disciplines, and educational levels. Longitudinal and experimental studies that combine self-reports with behavioral traces and independently evaluate task outputs would provide stronger evidence. The study did not examine cultural differences in students’ perceptions of GenAI. Although participants represented 15 nationalities, nationality was not treated as a measure of culture, and the study did not assess measurement invariance or compare the structural relationships across cultural groups. Differences in academic norms, prior exposure to GenAI, and interpretations of concepts such as trust and integrity may have influenced responses. Consequently, the pooled results should not be assumed to apply equally to every group represented in the sample. Some items across the constructs refer to similar practices, particularly checking AI outputs, evaluating ethical acceptability, and disclosing AI use. Although the cross-loadings and HTMT results support empirical separation in this sample, shared item content may have contributed to some associations. Future instrument refinement should test more sharply differentiated wording. The model tested selected theory-driven paths rather than every possible relationship. Comparisons among coefficients therefore apply only to predictors included in each specified outcome equation.

### 4.6. Implications and Future Research

The findings suggest that GenAI engagement should be addressed through differentiated practices rather than solely by encouraging AI use. Creation Behavior and Strategic Cognition were associated mainly with perceived productive and creative outcomes, whereas Verification Behavior and Critical Cognition were particularly relevant to Integrity and Trust Calibration. Community Behavior was associated with both Creativity and Integrity. Educators could therefore test structured creation–verification activities in which students generate content, evaluate its accuracy and limitations, document revisions, and justify decisions about reliance. Designers could similarly examine verification prompts, source-comparison functions, uncertainty indicators, and reflective feedback. These recommendations are design propositions derived from observed associations, not interventions validated by the present study. Future studies should replicate the measurement model and structural relationships in independently recruited samples from other universities, countries, disciplines, and educational levels. They should also test whether the measures operate comparably across groups before interpreting differences between them. Longitudinal or experimental designs, behavioral records, and independently assessed work products would help determine whether the observed associations persist and whether proposed practices improve outcomes. Future research should use sufficiently sized, independently recruited groups to examine cultural context, establish measurement comparability, and then test whether the observed relationships differ across groups.

## 5. Conclusions

This study examined seven affective, behavioral, and cognitive facets of GenAI engagement in a sample of 1250 students at eight universities in China. The findings showed outcome-specific associations: Verification Behavior was associated with perceived Output Quality, self-reported Integrity, and perceived Trust Calibration; Creation and Community Behavior were associated with perceived Creativity; and Critical Cognition was associated with Integrity and perceived Trust Calibration. Negative AI Affect was not associated with Integrity in the specified model. The model explained 17.6–25.7% of the variance across the four outcomes. Although its predictions performed better than the indicator-average benchmark, they did not show a statistically significant advantage over the linear-model benchmark. These results provide initial evidence for examining GenAI engagement through distinct facets in this sample. They do not establish causal effects, objective improvements in student work, or superiority over existing engagement or technology-adoption models. Replication and direct model comparisons are needed.

## Figures and Tables

**Table 1:** Sample Demographic Characteristics (*n* = 1250).

	Characteristic	*n*	%
**Gender**	Male	655	52.4
	Female	595	47.6
**Education**	Undergraduate	550	44.0
	Graduate	350	28.0
	PhD	132	10.6
	Prefer not to respond/unknown	218	17.4
**Discipline**	STEM	764	61.1
	Non-STEM	486	38.9
	**Total**	1250	100.0

**Table 2:** Construct-Level Descriptive Statistics and Reliability.

Construct	Code	Mean	SD	Range	Cronbach’s α	rho_A
Positive AI Affect	PAA	3.31	1.15	1–5	0.882	0.884
Negative AI Affect	NAA	2.82	1.14	1–5	0.869	0.871
Creation Behavior	CB	3.21	1.20	1–5	0.901	0.903
Verification Behavior	VB	3.32	1.15	1–5	0.887	0.889
Community Behavior	COM	3.22	1.15	1–5	0.873	0.875
Strategic Cognition	SC	3.27	1.16	1–5	0.887	0.898
Critical Cognition	CC	3.27	1.09	1–5	0.845	0.849
Output Quality	OQ	3.30	1.15	1–5	0.841	0.845
Integrity	IN	3.29	1.12	1–5	0.811	0.815
Creativity	CR	3.26	1.18	1–5	0.857	0.868
Trust Calibration	TC	3.16	1.19	1–5	0.862	0.875

Note. *N* = 1250. Means, SDs, and ranges are based on construct mean scores derived from five-point Likert items. α = Cronbach’s alpha; rho_A = Dijkstra–Henseler’s reliability coefficient. All reliability estimates exceed 0.70.

**Table 3:** Pairwise Correlations Between Study Variables.

Variable	1	2	3	4	5	6	7	8	9	10	11
Affective factors											
1. PAA	—										
2. NAA	−0.22 ***	—									
Behavioral factors											
3. CB	0.28 ***	−0.22 ***	—								
4. VB	0.01	−0.17 ***	0.25 ***	—							
5. COM	0.25 ***	−0.22 ***	0.25 ***	0.02	—						
Cognitive factors											
6. SC	0.26 ***	−0.18 ***	0.25 ***	0.26 ***	−0.05	—					
7. CC	−0.03	−0.20 ***	0.03	0.23 ***	0.03	0.31 ***	—				
Outcome factors											
8. OQ	0.28 ***	−0.19 ***	0.29 ***	0.33 ***	0.02	0.24 ***	−0.04	—			
9. IN	−0.02	−0.16 ***	0.00	0.35 ***	0.30 ***	−0.05	0.28 ***	0.27 ***	—		
10. CR	0.31 ***	−0.20 ***	0.38 ***	0.02	0.32 ***	0.25 ***	0.06 *	0.30 ***	0.32 ***	—	
11. TC	0.02	−0.22 ***	0.01	0.27 ***	0.09 **	0.02	0.34 ***	0.27 ***	0.28 ***	0.37 ***	—

Note. *N* = 1250. PAA = Positive AI Affect; NAA = Negative AI Affect; CB = Creation Behavior; VB = Verification Behavior; COM = Community Behavior; SC = Strategic Cognition; CC = Critical Cognition; OQ = Output Quality; IN = Integrity; CR = Creativity; TC = Trust Calibration. Values are Pearson product–moment correlations based on construct mean scores. Tests are two-tailed. * *p* < .05; ** *p* < .01; *** *p* < .001.

**Table 4:** Discriminant Validity. (**A**) Heterotrait–Monotrait Ratio (HTMT). (**B**) Fornell–Larcker Criterion.

**(A)**											
**Construct**	**1**	**2**	**3**	**4**	**5**	**6**	**7**	**8**	**9**	**10**	**11**
1. PAA	—										
2. NAA	0.256	—									
3. CB	0.313	0.244	—								
4. VB	0.021	0.194	0.285	—							
5. COM	0.287	0.257	0.281	0.026	—						
6. SC	0.289	0.202	0.284	0.290	0.062	—					
7. CC	0.042	0.236	0.041	0.266	0.043	0.361	—				
8. OQ	0.320	0.223	0.330	0.378	0.030	0.277	0.056	—			
9. IN	0.035	0.191	0.038	0.417	0.358	0.061	0.335	0.328	—		
10. CR	0.356	0.230	0.430	0.042	0.374	0.289	0.072	0.351	0.382	—	
11. TC	0.025	0.257	0.026	0.314	0.104	0.028	0.393	0.317	0.339	0.429	—
**(B)**											
**Construct**	**1**	**2**	**3**	**4**	**5**	**6**	**7**	**8**	**9**	**10**	**11**
1. PAA	**0.824**										
2. NAA	−0.224	**0.811**									
3. CB	0.279	−0.216	**0.847**								
4. VB	0.011	−0.172	0.256	**0.830**							
5. COM	0.252	−0.223	0.249	0.017	**0.814**						
6. SC	0.258	−0.179	0.257	0.261	−0.053	**0.830**					
7. CC	−0.034	−0.201	0.029	0.231	0.034	0.312	**0.786**				
8. OQ	0.277	−0.191	0.288	0.329	0.018	0.243	−0.043	**0.823**			
9. IN	−0.025	−0.161	−0.004	0.355	0.303	−0.047	0.280	0.272	**0.799**		
10. CR	0.311	−0.198	0.383	0.021	0.326	0.257	0.063	0.299	0.320	**0.836**	
11. TC	0.017	−0.225	0.008	0.277	0.092	0.018	0.342	0.269	0.286	0.368	**0.841**

Note A. PAA = Positive AI Affect; NAA = Negative AI Affect; CB = Creation Behavior; VB = Verification Behavior; COM = Community Behavior; SC = Strategic Cognition; CC = Critical Cognition; OQ = Output Quality; IN = Integrity; CR = Creativity; TC = Trust Calibration. All HTMT values were below the conservative threshold of 0.85 (Henseler et al., 2015). Bootstrapped 95% confidence intervals also supported discriminant validity: none included 1.00, and the largest upper confidence limit was 0.483. Note B. Bold diagonal values represent the square roots of the respective AVEs; off-diagonal values are latent-variable correlations. For every construct, the square root of AVE exceeded the absolute value of its correlations with all other constructs, satisfying the Fornell–Larcker criterion (Fornell & Larcker, 1981).

**Table 5:** Outer Loadings and Construct Reliability of Measurement Items by Domain.

Construct/Indicator	Outer Loading	ρc	AVE
Behavioral constructs			
Creation Behavior (CB)		0.927	0.718
CB1. I use AI to brainstorm or generate new ideas.	0.844		
CB2. I experiment with different prompts to see what AI creates.	0.833		
CB3. I iteratively refine AI outputs to improve them.	0.871		
CB4. I use AI to explore multiple possible solutions.	0.811		
CB5. I combine AI outputs with my own ideas to create something new.	0.875		
Verification Behavior (VB)		0.917	0.690
VB1. I fact-check AI-generated information before using it.	0.854		
VB2. I compare AI results with other reliable sources.	0.811		
VB3. I correct AI outputs when I notice inaccuracies.	0.803		
VB4. I make sure AI outputs align with ethical or academic standards.	0.835		
VB5. I evaluate AI content critically before submission or sharing.	0.848		
Community Behavior (COM)		0.908	0.663
COM1. I share useful AI prompts or insights with others.	0.804		
COM2. I discuss AI-generated ideas with my peers.	0.784		
COM3. I learn new ways of using AI from others.	0.792		
COM4. I give feedback to others about how they use AI.	0.849		
COM5. I acknowledge AI support in my shared work.	0.840		
Cognitive constructs			
Strategic Cognition (SC)		0.917	0.688
SC1. I plan how to use AI to reach specific goals.	0.784		
SC2. I organize my tasks to make AI assistance more effective.	0.839		
SC3. I think about which AI tool or prompt fits the task best.	0.861		
SC4. I adjust my strategy based on how AI responds.	0.878		
SC5. I reflect on how AI can support my learning or creativity.	0.781		
Critical Cognition (CC)		0.890	0.618
CC1. I question whether AI-generated results are fair or biased.	0.784		
CC2. I evaluate the sources AI uses to generate responses.	0.793		
CC3. I reflect on the ethical implications of using AI outputs.	0.820		
CC4. I think critically about how AI reaches its conclusions.	0.773		
CC5. I recheck the output produced by AI.	0.760		
Affective constructs			
Positive AI Affect (PAA)		0.914	0.679
PAA1. I feel excited when using AI tools for learning or creation.	0.824		
PAA2. Working with AI makes learning more enjoyable.	0.816		
PAA3. AI makes me curious to explore new topics or ideas.	0.841		
PAA4. I feel motivated to use AI to improve my performance.	0.799		
PAA5. Using AI puts me in a “state of flow” where time passes quickly.	0.841		
Negative AI Affect (NAA)		0.905	0.657
NAA1. I feel anxious when I rely too much on AI.	0.781		
NAA2. I sometimes feel uneasy about AI making decisions for me.	0.815		
NAA3. I get frustrated when AI does not understand my intent.	0.838		
NAA4. I worry that AI may lead me to make mistakes.	0.805		
NAA5. I feel uncomfortable trusting AI-generated information.	0.813		
Outcome constructs			
Output Quality (OQ)		0.894	0.678
OQ1. AI helps me produce higher-quality work.	0.860		
OQ2. AI improves the clarity and coherence of my output.	0.810		
OQ3. My work is more accurate when I use AI tools.	0.794		
OQ4. AI helps me achieve my intended goals more effectively.	0.828		
Integrity (IN)		0.876	0.638
IN1. I always disclose when I use AI tools in my work.	0.778		
IN2. I ensure AI-generated content is original and properly cited.	0.812		
IN3. I avoid misrepresenting AI-generated ideas as my own.	0.831		
IN4. I use AI in ways that uphold honesty and transparency.	0.773		
Creativity (CR)		0.903	0.699
CR1. AI inspires me to think in new and unconventional ways.	0.801		
CR2. I use AI to combine ideas in innovative ways.	0.869		
CR3. AI helps me generate more original ideas.	0.874		
CR4. I feel more creative when working with AI.	0.798		
Perceived Trust Calibration (TC)		0.906	0.707
TC1. I trust AI only after checking the accuracy of its outputs.	0.863		
TC2. I know when to rely on AI and when to verify its results.	0.772		
TC3. I can judge when AI outputs seem unreliable.	0.868		
TC4. I maintain a balanced level of trust toward AI systems.	0.857		

Note. ρc = composite reliability; AVE = average variance extracted. All standardized outer loadings exceeded 0.70 and were statistically significant at *p* < .001. Composite reliability values exceeded 0.70, and AVE values exceeded 0.50, supporting indicator reliability, internal consistency reliability, and convergent validity.

**Table 6:** Coefficients of Determination (R^2^).

Endogenous Construct	R^2^	R^2^ Adjusted
Creativity (CR)	0.257	0.255
Integrity (IN)	0.252	0.249
Output Quality (OQ)	0.208	0.205
Trust Calibration (TC)	0.176	0.174

Note. R^2^ represents the proportion of variance in each endogenous construct explained by its predictors.

**Table 7:** PLS-SEM Hypothesis-Testing Results.

Hypothesis	Structural Path	(β)	SE	(t)	(*p*)	95% CI	Decision
Affective engagement							
H1a	PAA → OQ	0.214	0.027	8.018	<.001	[0.162, 0.267]	Supported
H1b	PAA → CR	0.139	0.027	5.101	<.001	[0.085, 0.192]	Supported
H2a	NAA → IN	−0.004	0.025	0.174	.862	[−0.055, 0.045]	Not supported
H2b	NAA → TC	−0.137	0.026	5.343	<.001	[−0.190, −0.089]	Supported
Behavioral engagement							
H3a	CB → OQ	0.138	0.027	5.035	<.001	[0.083, 0.191]	Supported
H3b	CB → CR	0.240	0.027	8.888	<.001	[0.186, 0.292]	Supported
H4a	VB → OQ	0.269	0.026	10.374	<.001	[0.220, 0.322]	Supported
H4b	VB → IN	0.303	0.024	12.657	<.001	[0.258, 0.351]	Supported
H4c	VB → TC	0.191	0.026	7.215	<.001	[0.139, 0.243]	Supported
H5a	COM → CR	0.240	0.026	9.357	<.001	[0.190, 0.291]	Supported
H5b	COM → IN	0.290	0.024	11.837	<.001	[0.242, 0.338]	Supported
Cognitive engagement							
H6a	SC → OQ	0.083	0.029	2.882	.004	[0.027, 0.139]	Supported
H6b	SC → CR	0.172	0.026	6.536	<.001	[0.120, 0.224]	Supported
H7a	CC → IN	0.199	0.026	7.789	<.001	[0.149, 0.250]	Supported
H7b	CC → TC	0.270	0.025	10.915	<.001	[0.223, 0.319]	Supported

Note. (β) = standardized path coefficient; SE = bootstrapped standard error; CI = percentile bootstrap confidence interval; PAA = Positive AI Affect; NAA = Negative AI Affect; CB = Creation Behavior; VB = Verification Behavior; COM = Community Behavior; SC = Strategic Cognition; CC = Critical Cognition; OQ = perceived Output Quality; IN = Integrity; CR = Creativity; TC = Trust Calibration. Estimates were evaluated using 5000 bootstrap subsamples. A hypothesis was classified as supported when the estimated coefficient was in the hypothesized direction and its 95% bootstrap confidence interval excluded zero.

**Table 8:** (**A**) Out-of-Sample Predictive Assessment. (**B**) CVPAT Comparison with the Linear-Model Benchmark.

**(A)**								
**Outcome**	**Q^2^_Predict**	**RMSE**	**MAE**	**PLS Loss**	**IA Loss**	**Loss Difference**	***t***	***p***
Creativity	0.251	0.867	0.722	1.637	1.981	−0.345	10.056	<.001
Integrity	0.246	0.869	0.722	1.649	1.955	−0.307	9.686	<.001
Output Quality	0.200	0.895	0.743	1.702	1.970	−0.268	8.759	<.001
Trust Calibration	0.171	0.912	0.766	1.770	2.010	−0.240	7.989	<.001
Overall	—	—	—	1.689	1.979	−0.290	15.279	<.001
**(B)**								
**Outcome**	**PLS Loss**		**LM Loss**		**Loss Difference**		**(t)**	**(*p*)**
Creativity	1.637		1.661		−0.024		1.941	.053
Integrity	1.649		1.630		0.019		1.194	.233
Output Quality	1.702		1.708		−0.006		0.400	.689
Trust Calibration	1.770		1.780		−0.009		0.537	.591
Overall	1.689		1.695		−0.005		0.630	.529

Note A. Q^2^_predict = PLSpredict predictive-relevance statistic; RMSE = root mean squared error; MAE = mean absolute error; PLS loss = prediction loss generated by the PLS-SEM model; IA loss = prediction loss generated by the indicator-average benchmark. Negative loss differences indicate that the PLS-SEM model produced lower prediction error than the indicator-average benchmark. Note B. PLS loss = prediction loss generated by the PLS-SEM model; LM loss = prediction loss generated by the linear-model benchmark. Loss difference = PLS loss − LM loss; negative values descriptively favor PLS-SEM, whereas positive values favor the linear model. None of the differences were statistically significant at *p* < .05.

## Data Availability

The data supporting this study’s findings are available at the Harvard data repository (https://doi.org/10.7910/DVN/Q4ZZP2) and can be requested from the corresponding author.

## Appendix A

**Table A1:** Detailed Participant Demographic Characteristics (*N* = 1250).

Characteristic	Category	*n*	%
Gender	Male	655	52.4
	Female	595	47.6
Education Level	Undergraduate	550	44.0
	Graduate (Master’s)	350	28.0
	Doctoral (PhD)	132	10.6
	Prefer not to respond/Unknown	218	17.4
Discipline	STEM	764	61.1
	Non-STEM	486	38.9
Nationality	Chinese	470	37.6
	Pakistani	320	25.6
	Yemeni	60	4.8
	Egyptian	55	4.4
	Japanese	45	3.6
	Bangladeshi	45	3.6
	Indian	40	3.2
	Ethiopian	35	2.8
	Russian	30	2.4
	Cameroonian	28	2.2
	Ugandan	27	2.2
	Indonesian	25	2.0
	Tajikistani	25	2.0
	Iranian	23	1.8
	Azerbaijani	22	1.8
Total Participants		1250	100.0

## Appendix B

**Table A2:** Indicator-Level PLSpredict Results.

Indicator	Q^2^_predict_	PLS RMSE	LM RMSE	PLS MAE	LM MAE
CR1	0.130	1.322	1.329	1.129	1.126
CR2	0.208	1.260	1.275	1.065	1.069
CR3	0.217	1.232	1.236	1.044	1.033
CR4	0.142	1.300	1.313	1.102	1.110
IN1	0.148	1.289	1.291	1.091	1.087
IN2	0.175	1.268	1.259	1.070	1.053
IN3	0.180	1.282	1.261	1.086	1.059
IN4	0.124	1.297	1.295	1.101	1.097
OQ1	0.164	1.297	1.301	1.102	1.089
OQ2	0.123	1.297	1.297	1.107	1.103
OQ3	0.120	1.318	1.320	1.124	1.119
OQ4	0.135	1.306	1.310	1.107	1.097
TC1	0.143	1.300	1.308	1.101	1.101
TC2	0.077	1.356	1.368	1.174	1.176
TC3	0.150	1.311	1.303	1.126	1.101
TC4	0.108	1.354	1.356	1.167	1.159

Note. Q^2^_predict_ = indicator-level predictive-relevance statistic; PLS = PLS-SEM prediction; LM = linear-model benchmark; RMSE = root mean squared error; MAE = mean absolute error. Positive Qpredict2 values indicate predictive relevance relative to the naïve mean-based benchmark. Lower RMSE or MAE values indicate lower prediction error. PLS-SEM’s comparative performance against the LM benchmark varied across indicators and error measures.
